# Pricing of HPV vaccines in European tender-based settings

**DOI:** 10.1007/s10198-018-0996-9

**Published:** 2018-07-26

**Authors:** Venetia Qendri, Johannes A. Bogaards, Johannes Berkhof

**Affiliations:** 1 Department of Epidemiology and Biostatistics, VU University Medical Center, PO Box 7057, MF F-wing ST, 1007 MB Amsterdam, The Netherlands; 2 National Institute for Public Health and the Environment, Bilthoven, The Netherlands

**Keywords:** HPV, Vaccine, Price, Tender, Procurement, Europe

## Abstract

**Background:**

Vaccine price is one of the most influential parameters in economic evaluations of HPV vaccination programmes. Vaccine tendering is a cost-containment method widely used by national or regional health authorities, but information on tender-based HPV vaccine prices is scarce.

**Methods:**

Procurement notices and awards for the HPV vaccines, published from January 2007 until January 2018, were systematically retrieved from the online platform for public procurement in Europe. Information was collected from national or regional tenders organized for publicly funded preadolescent vaccination programmes against HPV. The influence of variables on the vaccine price was estimated by means of a mixed-effects model.

**Findings:**

Prices were collected from 178 procurements announced in 15 European countries. The average price per dose for the first-generation HPV vaccines decreased from €101.8 (95% CI 91.3–114) in 2007 to €28.4 (22.6–33.5) in 2017, whereas the average dose price of the 9-valent vaccine in 2016–2017 was €49.1 (38.0–66.8). Unit prices were, respectively, €7.5 (4.4–10.6) and €34.4 (27.4–41.4) higher for the 4-valent and 9-valent vaccines than for the 2-valent vaccine. Contract volume and duration, level of procurement (region or country), per capita GDP and number of offers received had a significant effect on vaccine price.

**Interpretation:**

HPV vaccine procurement is widely used across Europe. The fourfold decrease in the average tender-based prices compared to list prices confirms the potential of tendering as an efficient cost-containment strategy, thereby expanding the indications for cost-effective HPV vaccination to previously ineligible target groups.

**Electronic supplementary material:**

The online version of this article (10.1007/s10198-018-0996-9) contains supplementary material, which is available to authorized users.

## Introduction

Oncogenic types of human papillomavirus (HPV) are sexually transmitted and are nowadays recognised as major risk factors for cervical, vulvar, vaginal, anal, and oropharyngeal cancers in women, and anal, penile, and oropharyngeal cancers in men [1]. Three vaccines are registered for prevention of HPV infections: the bivalent vaccine (Cervarix^®^, GSK) and the quadrivalent vaccine (Gardasil^®^, Merck & Co) have been available since 2007, while the nonavalent vaccine (Gardasil9^®^, Merck & Co) has become available since 2016 [2–4]. All vaccines protect against the high-risk types HPV16 and 18, responsible for the majority of the HPV-related cancers. The quadrivalent vaccine also protects against the low risk types HPV6 and 11, accounting for about 90% of genital warts [3], and the nonavalent vaccine protects against 5 more oncogenic types (31, 33, 45, 52 and 58) [4]. In Europe, all vaccines are licensed for use in females and males, whereas in the US, only the quadrivalent and the nonavalent are available for both [2–5].

Most countries have focused on vaccination of females, as they experience the greatest HPV-related disease burden [6]. However, the exclusion of preadolescent boys in national immunization programmes (NIP) has caused equity concerns, because HPV vaccines have the potential to prevent cancers in both men and women [7–9]. Girls-only programmes with high vaccine uptake may partly protect heterosexual males via a reduced exposure to HPV infection [10–12], but in many countries, vaccine uptake among girls has been too low to achieve complete indirect protection to men [13]. Inclusion of boys in NIP may be an appealing option for improving the health impact of HPV vaccination programmes, albeit cost-effectiveness analyses have not yielded consistent results: some were positive [14–17], but most recommended against sex-neutral vaccination [9, 18–25]. Commonly cited obstacles are the prohibitive list prices of the HPV vaccines [26–28] and the multiple doses recommended for achieving sufficient protection [2–4]. However, despite the controversy on its economic feasibility, sex-neutral vaccination is endorsed in an increasing number of jurisdictions [7, 9].

To better understand policy differences between countries, a closer look at the price of the HPV vaccines in NIP is required, given its key role in the health-economic evaluations of HPV vaccination. HPV vaccine prices in national vaccination programmes can be substantially lower than their respective list prices. To control the continuously rising pharmaceutical expenditure, governments across countries look for effective mechanisms to yield savings to the health care budgets [29, 30]. For vaccination expenditure, a commonly used cost-containment mechanism is tendering: health authorities use their purchasing power and the competition in the market of the vaccines to perform procurement procedures. This drives down the cost and in that way enhances efficiency and sustainability of a programme. Low recourse settings can procure vaccines through external procurement agents such as UNICEF’s Supply Division and the Pan American Health Organization (PAHO) Revolving Fund. In contrast, middle- and high-income countries most often opt for direct public procurement of bulk quantities [31, 32], as is the case in Europe [33]. In 2013, according to a WHO survey, 41 vaccines were procured in 30 European member states with about half of the member states having already established procurement procedures for the new HPV vaccines [34]. Experience with older vaccines, such as hepatitis B vaccines (HBV), suggests that procurement mechanisms may lead to strong price reductions over time [32, 35, 36]. For the HPV vaccines, to our knowledge, the only available evidence supporting this premise is a WHO report published in 2016, but with limited data [35, 36], and a study from Italy revealing that HPV vaccine prices in the majority of the Italian regions dropped to almost half of their introductory prices within only 2 years of tendering [37].

Paucity on tender-based vaccine prices (i.e., the winning prices at the procurement process), especially for new vaccines, has recently been highlighted as an obstacle to vaccine accessibility [34, 35]. In several countries, the confidential nature of procurement agreements impedes transparency in vaccine pricing, but some countries provide access to procurement based information. Our study has two main objectives. First, we aimed to collect information on tender procedures for the HPV vaccines in European NIPs. Second, we aimed to identify variables that are associated with HPV vaccine pricing in the different tender-based settings.

## Methods

### Data collection via Tenders Electronic Daily

**Fig. 1:**
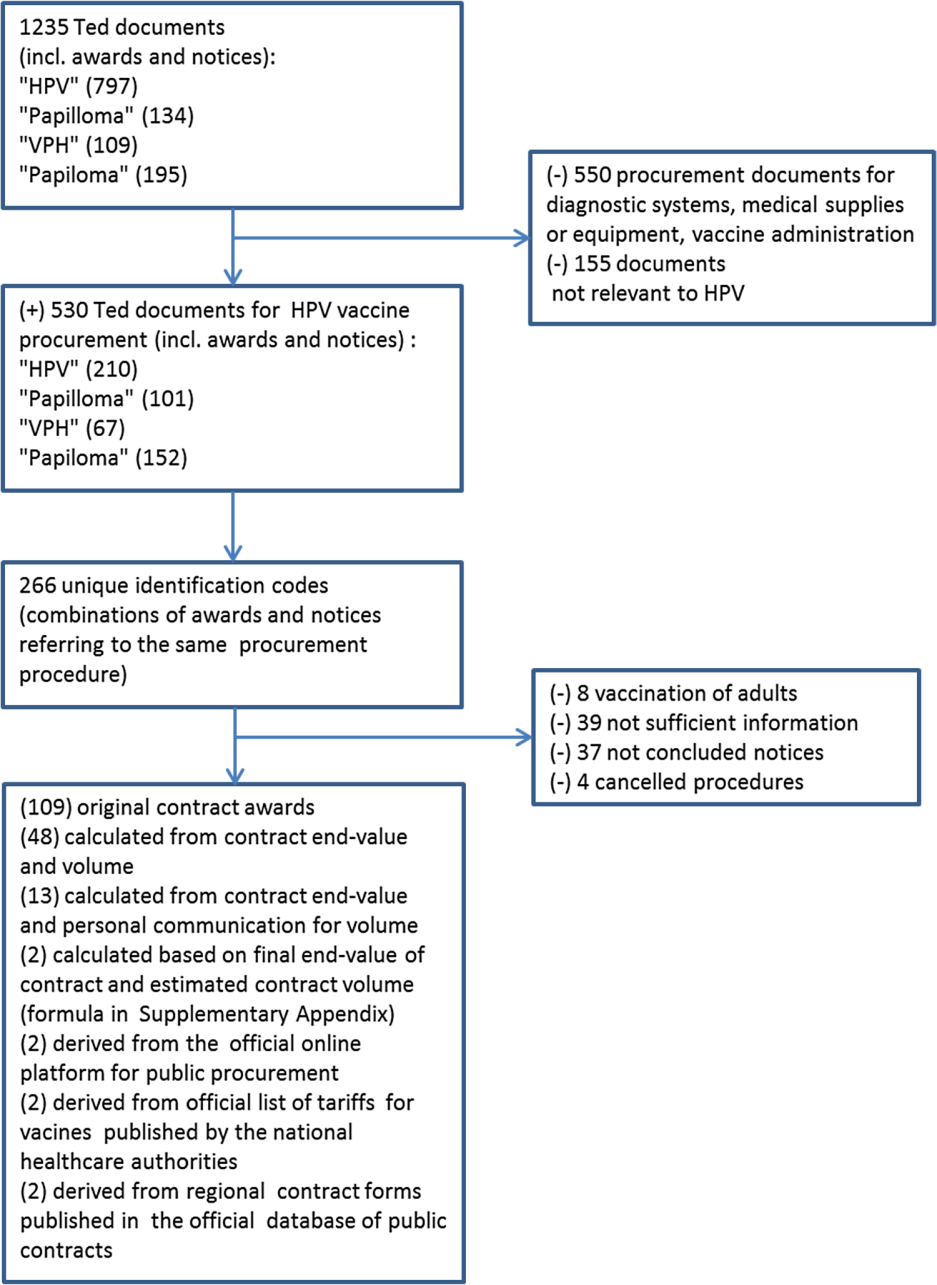
Diagram of data collection process for the unit prices of the HPV vaccines

We conducted a search on the Tenders Electronic Daily (TED), the online version of the “Supplement to the Official Journal of the European Union” dedicated to public procurement in Europe [38]. This platform provides free electronic access to calls for tenders published by EU institutions, agencies, and other bodies. In addition, it gives the opportunity to identify and sort procurement notices and awards by country, region, and business sector. We searched the platform’s archives from January 2007 up to January 2018 using the search terms “HPV”, “papilloma”, “VPH”, and “papiloma” (Fig. 1). The last two terms are used in Spanish and Portuguese. Given that the documents stored in the original language contained more information than English versions, we collected all documents in their original language and applied online tools for translation.

Initially, this process led to the identification of 19 European countries using procurement procedures for the HPV vaccines: Austria, Belgium, Croatia, Denmark, Estonia, France, Hungary, Iceland, Italy, Latvia, FYR Macedonia, The Netherlands, Norway, Poland, Portugal, Spain, Slovenia, Sweden, and the UK. Most European countries organize vaccine tendering at national level, but in some countries (namely, Belgium, France, Hungary, Italy, Poland, and Spain) procurement is conducted by the regional public health authorities. In our data collection, we included tender-based information from both settings, regional or national, and we focussed on procurement procedures for routine vaccination of preadolescent girls or boys. Details about the tender awards can be found in the Supplementary Appendix.

### HPV vaccine price

Figure 1 presents the process of the data collection for the unit prices of the vaccines. We matched contract notices with the respective contract awards from the TED website and reviewed the documents to identify unit prices of the HPV vaccines. The standardized award notices from the TED website most often stated only the total end-value of the contract (VAT included or excluded) reflecting the final agreement on the procurement of a specified volume. When the volume was available in the TED documents, calculation of the tender-based price per dose of the vaccine was straightforward. When information on both tender-based unit prices and contract volume was missing, we tried to retrieve the original contract agreement via the website of the contracting authority using the identification code and name of the contract. In case we were still unable to locate the original contract, we contacted the person/authority responsible for the vaccine procurement procedure. Last, if the responsible person/authority did not respond to our request, we used country-specific public information on HPV vaccine coverage, size of birth cohort, year-specific dosing schemes, and number of contract years to estimate the contract volume (Supplementary Appendix).

Different currencies were adjusted using the World Bank’s average annual exchange rates for 2015 (Supplementary Appendix). In addition, we stored information on the following parameters: the type of the vaccine that was awarded; the duration of the contract; the contract volume, defined as the agreed number of doses per year; the number of offers received in the procurement procedure (2 versus 1); the award selection criterion (dichotomized; price versus price and other criteria); and the level of the vaccine procurement (national versus regional). To account for differences in procurement outcomes when delivering for a new compared to an established HPV vaccination programme, we also stored a dichotomous variable which took the value of 1 when the procurement in a country or region was the first procurement (in our data set) for the specific country or region and 0 otherwise. Besides, we obtained country-specific information on per capita gross domestic product (GDP) in 2015.

### Statistical analysis

Our data set contains multiple observations over time for each country/region; thus, we utilized a linear mixed-effects model [39]. The tender-based unit price was modelled as a natural cubic spline function over time [40]. Time was calculated as the number of days between the contract award date and Jan 1st 2007. The coefficients of the cubic spline were evaluated by the likelihood ratio test [41]. Change over time was also evaluated for the stored vaccine and contract features by linear mixed-effects models for continuous outcomes and generalized linear mixed-effects models for binary outcomes. As a next step, the stored vaccine and contract features and per capita GDP were added as covariates in a multivariable model for vaccine price as a function of time. The proportion of the total contract price variation explained by the covariates is a function of the total variance from a model with only a random intercept and the total variance in a mixed regression model including covariates (Supplementary Appendix). All statistical analyses were performed in R version 3.4.2.

## Results

### Contract characteristics

We identified 178 procurement awards with complete information on the per-dose price of the HPV vaccines (Fig. 1). Prices were available for 15 out of the 19 identified European countries with self-procurement procedures for the HPV vaccines (Supplementary Appendix). The average per capita GDP level of these countries was €28,000 and varied from €18,400 in Croatia to €50,900 in Norway. In Denmark, France, and the UK, information on the procurement procedures was not made available to the public, and in FYR Macedonia, we were unable to get any follow-up information on the announced procurement. For all countries in our data set, information from at least two consecutive contracts was available (varied from 2 to 6), with the exception of Estonia and Norway. In Norway, information was publicly available only for the tender of 2009, but not for subsequent tenders, and in Estonia, HPV vaccination of girls in the national vaccination schedule was introduced only in 2017.

**Fig. 2:**
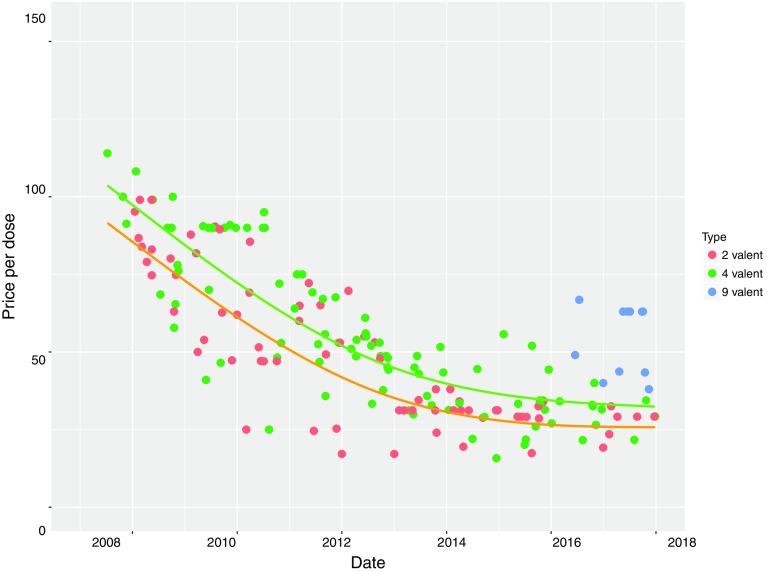
Tender-based per-dose prices of the HPV vaccines stratified by the awarded type of the vaccine versus the date of the contract award. Green and orange lines represent model fit for the 4-valent and 2-valent vaccines, respectively. Data fitting is conducted with a mixed model with random intercepts at the country/region level and a cubic spline of the contract date

Table 1 summarizes the descriptive characteristics of the contract awards. Contracts for the 2-valent and 4-valent vaccines were roughly equally distributed over the time period of the analysis, apart from 2007, when only the 4-valent vaccine was registered. For the 9-valent vaccine, tendering information was limited to the last 2-year period, as the vaccine was introduced 2016. In more than 80% of the tenders, both vaccine manufactures responded to tender calls (open tenders). The mean volume of contracts was 38,000 doses per year, but varied from 20 doses per year (regional level) to 180,000 (national level) doses per year. Furthermore, contract volume significantly decreased over time by about 1800 doses per year (95% confidence interval (CI) 1100–3500) since vaccine introduction in 2007 (Figs. 2–4 in Supplementary Appendix). Contract duration remained constant over time (*p* = 0.17). 14% of the agreements concluded on a contract period of 3 or 4 years, while the majority of agreements were made on a yearly basis with possibility of extension. The pattern for the award criteria did not change over the analysis period (*p* = 0.67) and about one-third of the countries/regions in our data set used the best or the lowest price as their key criterion for awarding the tender. In most occasions, quality and the ability to supply were also explicitly mentioned, although weighed less than the price criterion (Supplementary Appendix).

**Table 1:** Characteristics of the contract awards

	Total contracts (2007–2017)	2007	2008	2009	2010	2011	2012	2013	2014	2015	2016	2017
Type of the vaccine												
2-valent	71	–	11	9	7	8	5	9	9	7	1	5
4-valent	96	3	10	10	8	11	15	9	6	13	8	3
9-valent	11	–	–	–	–	–	–	–	–	–	3	8
Total	178	3	21	19	15	19	20	18	15	20	12	16
Contract volume												
Mean number of doses per year × 1000 (min.–max.)	35.9 (0.02–180)	11 (0.2–30)	38 (0.5–111)	40 (1.6–125)	46 (0.1–150)	32 (0.8–120)	25 (0.02–180)	42 (0.3–151)	45 (1.5–110)	30 (0.02–133)	35 (4–101)	34 (0.03–145)
Contract duration												
1 year	105	3	15	12	9	12	16	5	8	11	6	8
2 years	45	–	3	5	3	5	4	8	4	5	4	4
3 years	19	–		2	2	1	–	3	1	4	2	4
≥ 4 years	9	–	3	–	1	1	–	2	2	–	–	–
Number of offers received												
1 offer	36	2	3	2	0	1	3	2	2	6	5	10
2 offers	142	1	18	17	15	18	17	16	13	14	7	6
Award criteria												
Price	61	2	7	6	5	5	4	8	5	7	4	8
Price and other	117	1	14	12	9	14	16	10	10	13	8	8
Level of procurement												
National	41	–	1	4	1	2	3	10	6	4	5	10
Regional	137	3	20	15	14	17	17	8	9	16	7	6
First procurement for each country/region in data set	50	3	18	7	5	6	3	1	0	5	0	2
Income level (per capita GDP)												
< 30,000	118	2	14	14	9	15	17	11	9	8	6	7
> 30,000	60	1	7	5	6	4	3	7	6	12	6	9

### Tender-based HPV vaccine prices

Unit prices are plotted in Fig. 2 by the type of vaccine awarded. The average tender-based price per dose of the first-generation HPV vaccines decreased from €101.8 (91.3–114) in 2007 to €28.4 (22.6–33.5) in 2017, whereas the average dose price of the 9-valent vaccine in 2016–2017 was €49.1 (38.0–66.8). Curves show the decline in the average unit prices over time, as estimated by the natural cubic spline function for the 2-valent and 4-valent vaccines separately. Differences in annual decline between the 2-valent and 4-valent vaccines were not significant (*p* = 0.65).

**Fig. 3:**
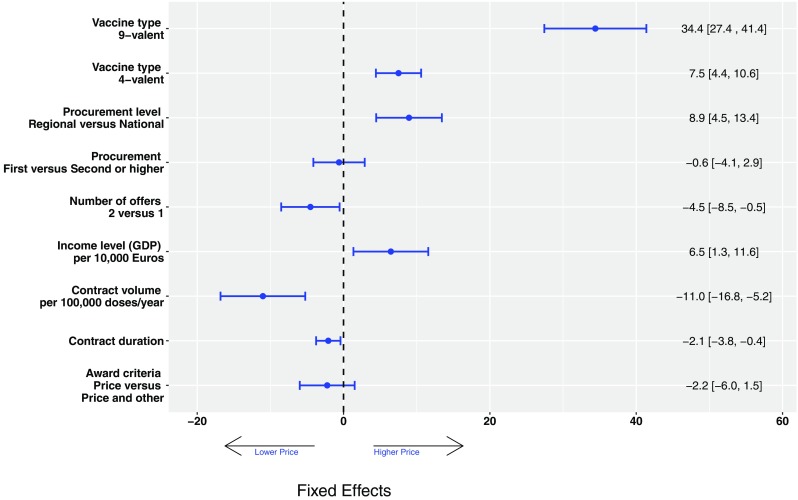
Fixed effects estimates from a multivariable mixed model with a random intercept at the country/region level

Estimates of the fixed effects are shown in Fig. 3. Vaccine type, contract volume, and duration, level of the vaccine procurement, per capita GDP and number of offers received were significantly associated with the tender-based prices of the HPV vaccines. Specifically, for the 4-valent and 9-valent vaccines tender-based unit prices were, respectively, €7.5 (4.4–10.6) and €34.4 (27.4–41.4) higher than for the 2-valent vaccine. Contract volume was associated with a decrease in the per-dose price of €11.0 (5.2–16.8) per 100,000 doses per year, whereas organising procurement procedures at regional rather than national level resulted in higher unit price of €8.9 (4.4–13.6) per dose. An increase of €10,000 in the per capita GDP level was also associated with an increase in the tender price of the HPV vaccine of €6.5 (1.3–11.6) per dose. Receiving bids from both manufacturers lowered the unit price by €4.5 (0.5–8.5) compared to procurements that received only one offer. Finally, increasing the contract duration and defining vaccine price as the award criterion (compared to price and other) had minor negative effects of €2.1 (0.4–3.8) and €2.2 (− 1.5 to 6.0) on the vaccine price, whereas the impact of first versus second or later HPV procurement procedure was close to zero. Different interaction effects between covariates were investigated, but significant interaction effects on vaccine price were found only for contract volume and time (estimate 2.1, 95% CI 0.5–3.5) and level of vaccine procurement (regional versus national) with vaccine type (estimate − 15.6, 95% CI − 23.5 to − 7.8, for the 4-valent and − 32.2, 95% CI − 47.9 to − 16.8., for the 9-valent). The proportion of the total explained variance in prices was 68% when including only the time function and type of vaccine and increased to 80% when adding other covariates.

## Discussion

This study presents systematically collected information on HPV vaccine unit prices in European tender-based settings from 2007 up to and including 2017. Our findings indicate that procurement procedures for the HPV vaccines are widely used across Europe, with 19 European countries announcing procurement procedures on the online platform for public procurement in Europe. Moreover, our analysis reveals that, setting-specific variation notwithstanding, strong price reductions have been achieved over time for the HPV vaccines since vaccine introduction in 2007, thereby increasing the economic efficiency of HPV vaccination.

We observed a decrease in the tender-based price from over 100 to below 30 for first-generation vaccines during a 10-year period and an average price of €50 for the new nonavalent vaccine. The fourfold reduction in tender-based prices compared to list prices of the first-generation HPV vaccines confirms the potential of tender procedures as a tool to ensure affordable prices. This, in turn, may increase the scope for HPV vaccination. In many countries, the decision for girls-only vaccination programmes was motivated by the high burden of cervical cancer. This burden was considered to justify considerable expenditure on HPV vaccination, stemming from high HPV vaccine list prices and the multiple doses required to achieve optimal protection [7]. This principle was adopted by the majority of the health-economic evaluations around the time of the vaccine introduction, which endorsed girls-only vaccination, but advised against sex-neutral vaccination as a consequence of the presumed high cost of intervention [9, 18, 20–22, 24–27]. Later, economic evaluations reported on average fourfold reduced incremental cost-effectiveness ratios of sex-neutral relative to girls-only vaccination by accounting for other health outcomes than only cervical disease [42]. However, the ratios often remained above acceptable thresholds for cost-effective interventions. The results of the current analysis indicate that the incremental cost-effectiveness ratios of sex-neutral vaccination in national immunization programmes may be reduced by a similar factor by considering outcomes from established tender procedures. Implementation of reduced dosing schemes further reduces the cost of vaccination improving the cost-effectiveness of sex-neutral vaccination. Indeed, recent cost-effectiveness studies that accounted for all HPV-related outcomes as well as the observed reductions in vaccination cost have reported favourably on the cost-effectiveness of sex-neutral vaccination [14, 17].

The procurement outcome was strongly associated with calendar time. A possible explanation for this association is that the long experience with HPV vaccine tendering drives down the tender-based vaccine price. However, our regression analyses indicated that procuring the HPV vaccine for the first time versus the second or later time had a nearly negligible influence on the vaccine price. Two other explanations that could not be examined in our current analyses are the following: first, information spreading about low tender-based prices in other countries may have a downward effect on the vaccine price. Second, the high vaccine price together with a rapid, widespread adoption of HPV vaccines suggests high returns on investment in the first years after registration. This may have encouraged manufacturers to allow for vaccine price reduction over time to maintain or increase market shares.

Although time explained most of the variation in the tender-based prices of the HPV vaccines, a number of other variables were identified that influence the outcome of the procurements. First, tenders awarding the 4-valent and 9-valent vaccines resulted in respective unit prices which were about €8 and €30 higher than the award prices of the 2-valent vaccine. It is worth mentioning that estimated tender-based price differences between the first-generation vaccines are lower than the price differences that are justified according to health-economic analyses [43, 44], whereas the reverse seems to hold when comparing the tender-based prices of the 4-valent and 9-valent vaccines [45]. Second, our estimates revealed that centralising vaccine procurements and increasing the contract volume and duration may substantially decrease the agreed vaccine price. This finding is consistent with the law of supply and demand. Efforts implementing this theory and benefiting from economies of scales include the PAHO Revolving Fund and the Gulf Cooperation Council (GCC) [32]. Nonetheless, a caveat needs to be taken into account when considering this premise: achieving low prices via highly centralised procurements with long-lasting agreements runs the risk to erode market competition by forcing some competitors to withdraw from the market [46–49]. This unintended market failure may lead to a future price increase [46–49]. Third, the analysis shows that tenders designed to allow both manufacturers to bid can achieve a better price than direct negotiation procedures with only one manufacturer. That is in alignment with the previous analyses strengthening the importance of competition augmentation in tendering procedures even when the number of competitors is minimal [49, 50]. This finding further highlights that economic sustainability of HPV vaccination requires the 2-valent vaccine to maintain a substantial market share, given that the 4-valent and 9-valent vaccines are produced from the same manufacturer [51]. Finally, results show a positive association between the country-specific income level and the agreed single price per HPV vaccine. This agrees with tiered pricing, a strategy employed by vaccine manufacturers which requires wealthier countries to be charged more for the same vaccine than poorer countries [34, 35]. The previous analysis from the WHO indicated that while prices for hepatitis B and pneumococcal vaccines tended to be adjusted according to the country’s income level, no adjustments were made for the HPV vaccines [34, 35]. Although we observed some adjustment in HPV pricing according to the country’s income level, the WHO report suggests that compared to other vaccines there is still a lot to be gained in terms of affordable pricing for countries with relatively low income levels.

Our data indicated a slight reduction over time in the number of the HPV vaccines. Several reasons may explain the observed decrease in contract volume. First, since 2014, many national HPV vaccination programmes have switched from 3-dose to 2-dose regimes for preadolescent vaccination, indicating a need for fewer vaccine doses. Second, in many national programmes, HPV vaccination achieved lower vaccine uptake than originally anticipated at the time of the vaccine initiation. Furthermore, in several European countries, HPV vaccine uptake dropped over time with some countries experiencing gradual reductions, such as Italy and The Netherlands, while others experiencing more rapid declines, such as Denmark. A last potential reason for the observed reduction in contract volume may be the fact that many countries introduced catch up campaigns during the first years of the vaccine administration indicating a need for a higher volume at the beginning of the programme compared to the volume needed in the following years.

A limitation of the present study is that we may have not adequately captured all potential factors influencing tender-based vaccine prices in the different settings. Contractual terms include some of the variables expected to have a potential impact on the final unit price of the procurement, such as insurance fees, packaging options, and transportation costs [34]. Organization of HPV vaccination programmes may also influence procurement outcome as well-organized programmes carry a lower risk for both the health authorities and the vaccine manufacturer. Another potential limitation is that for some of the contract awards, we employed quantitative methods to calculate the tender-based unit price of the vaccines (2 tenders) and the number of doses that concerned the specific award (15 tenders). Finally, we searched via the EU institutions’ eProcurement platform, i.e., TED website, as our primary source of information and did not consider procurements that were not registered.

To conclude, our findings confirm that tendering is an effective tool for the procurement of the HPV vaccines leading to more affordable pricing in Europe. This paper aimed to reduce information asymmetry between national immunization programme managers, vaccine purchasers, and the research community in the field of HPV, by promoting transparency in pricing of the HPV vaccines in European tender-based settings. Considering the limited evidence in the field of tender-based vaccine pricing, a similar exercise would be worthwhile for more vaccines, especially for recently introduced vaccines with high dose prices. Such efforts can help reduce prices in EU countries and may in that way contribute to the introduction of HPV vaccination in resource-limited countries and to the extension of a girls-only programme to other target groups.

## Electronic supplementary material

Below is the link to the electronic supplementary material.

Supplementary material 1 (DOCX 179 KB)
