# Supplementary material for: Pricing of HPV vaccines in European tender-based settings
Source: Eur J Health Econ. 2018 Jul 26;20(2):271–80. doi: 10.1007/s10198-018-0996-9 (PMC6439217; doi:10.1007/s10198-018-0996-9)
Supplement: Supplementary file 1 — Supplementary material 1 (DOCX 179 KB) [file 10198_2018_996_MOESM1_ESM.docx]

**Pricing of HPV vaccines in European tender-based settings**

# Supplementary Appendix

# Supplementary Methods

## Data collection

When the contract end-value and volume of the agreement were available in the TED documents, calculation of the tender-based price per-dose of the vaccine was straightforward: we divided contract end-value by volume. When information on both tender-based unit prices and contract volume was missing, we tried to retrieve the original contract agreement via the website of the contracting authority using the identification code and name of the contract (first column of table 5). In case we were unable to locate the original contract, we contacted the person/authority responsible for the vaccine procurement procedure (in the table below indicated as personal communication). If the responsible person/authority did not respond to our request and tendering was conducted at the national level, we used country-specific public information on HPV vaccine coverage, size of birth cohort, year-specific dosing schemes and number of contract years and estimated the contract volume with the following formula:

$Eq. 1 Contract Volume=Births/Year\times Vaccine coverage\times Vaccination Schedule\times Contract Duration$

Here, number of births per year was calculated as the average number of births from 2014-2016, extracted from national statistics. The vaccination schedule is 2 if a 2-dose schedule is used and 3 otherwise. Information on country-specific vaccine coverage was extracted from scientific literature or from reports prepared for the performance of national vaccination schedules.

Subsequently, the unit price was calculated using the following formula:

$$Eq. 2 Unit price=\frac{Total end value of contract}{Contract Volume}$$

Equation 1 was also used to estimate the contract volume in countries where the tender-based unit price was available but no information was available on the contract volume. For regional tenders, when the tender-based unit price was identified but contract volume was not available, we estimated the contract volume either by dividing the contract end-value by the unit price of the vaccine or by using information on contract volume and duration from previous or subsequent tenders in the same region.

## Total explained variance

$${var}_{X}=1- \frac{\sigma_{X}^{2}}{\sigma_{null}^{2}}$$

Where$\boldsymbol{\sigma}_{\boldsymbol{null}}^{\boldsymbol{2}}$ the total variance in the null model: $\boldsymbol{\sigma}_{\boldsymbol{null}}^{\boldsymbol{2}}\boldsymbol{=}\boldsymbol{\sigma}_{\boldsymbol{u}}^{\boldsymbol{2}}\boldsymbol{+}\boldsymbol{\sigma}_{\boldsymbol{\varepsilon}}^{\boldsymbol{2}}$**,** with $\boldsymbol{\sigma}_{\boldsymbol{u}}^{\boldsymbol{2}}$ the between-country/region variance and $\boldsymbol{\sigma}_{\boldsymbol{\varepsilon}}^{\boldsymbol{2}}$ the within-country/region variance. Similarly, $\boldsymbol{\sigma}_{\boldsymbol{X}}^{\boldsymbol{2}}$ is the total variance in a mixed regression model including variables $\boldsymbol{X}$.

| Supplementary Tables and Figures **Table 1. Data collection** | | | | | | | | | | | | | |
| --- | --- | --- | --- | --- | --- | --- | --- | --- | --- | --- | --- | --- | --- |
| **Country/**  **Region** | **Reference** | **Date of contract Award** | **Type of vaccine** | **Value of Contract** | **Value of Contract in Euros** | **Contract Duration** | **Persons to be vaccinated** | **Total doses** | **Doses**  **/Year** | **Contract Authority** | **Number of offers Received** | **Award Criteria** | **Price per Dose**  **(VAT Excl.)** |
| Austria^[[1]](#footnote-1)^ | **2016/S 146-263415**  **(Source TED)** | 15.07.2016 | 9-valent | - | 20,185,290 | 3 years | 50,400 per year  (Estimated 84000*0,6) | 302,400 (Estimated)  (2-dose) | 100,800 | Federal Procurement Agency (BBG, Bundesbeschaffung GmbH, [www.bbg.gv.at](http://www.bbg.gv.at)) | 1 | 1. Price | 66.8 |
| Austria1 | **2013/S 233-404152**  **(Source TED)** | 19.11.2013 | 4-valent | - | 15,600,000 | 2 years | 50,400 per year  (Estimated 84000*0,6) | 302,400 (Estimated)  (3-dose) | 151,200 | Federal Procurement Agency (BBG, Bundesbeschaffung GmbH, [www.bbg.gv.at](http://www.bbg.gv.at)) | 1 | 1. Price (50) 2. Quality(50) | 51.6 |
| Belgium (Flanders) | **2014/S 098-171262**  **(Source TED)** | 28.04.2014 | 2-valent | - | 6,826,400 | 4 years | 35,000 per year | 280,000 | 70,000 | Vlaams Agentschap Zorg en Gezondheid, [www.zorg-en-gezondheid.be](http://www.zorg-en-gezondheid.be) | 2 | 1. Price (40)  2. Intrinsic quality (40)  3. Fitness to general vaccination program (10)  4. Stability in cold chain problems (10) | 19.5 |
| Belgium (Flanders) | **2010/S 171-261374**  **(Source TED & Personal Communication)** | 11.08.2010 | 4-valent | - | 7,631,470 | 4 years | - | 385,000 | 105,000 | Vlaams Agentschap Zorg en Gezondheid, [www.zorg-en-gezondheid.be](http://www.zorg-en-gezondheid.be) | 2 | 1. Price (40)  2. Intrinsic quality (40)  3. Fitness for general vaccination program. (10)  4. Stability in cold problems (10) | 25.0 |
| Croatia | **2017/S 239-495918**  **EVV 01/17**  **(Source Ted,** [www.hzjz.hr](http://www.hzjz.hr), [www.eojn.nn.hr](http://www.eojn.nn.hr) **& Personal Communication**) | 20.04.2017 | 9-valent | 6,650,000 HRK  (1 HRK = 0.131469675 EUR) | 874,273 | 1 year | - | 20,000 | 20,000 | Croatian Institute for Public Health (Hrvatski zavod za javno zdravstvo, [www.hzjz.hr](http://www.hzjz.hr)) | 1 | 1. Price (40)  2. Expected reduction in the risk of precancerous intraepithelial lesions of the cervix and the high degree of invasive cervical cancer (35)  3. Acceptance of the vaccine in 2016 (25) | 43.7 |
| Croatia | **TED**  **EVV 26/16**  **(Source** [www.hzjz.hr](http://www.hzjz.hr), www. eojn.nn.hr) | 19.12.2016 | 4-valent | 1,195,650 HRK  (1 HRK = 0.131469675 EUR) | 157,191 | 1 year | - | 5,000 | 5,000 | Croatian Institute for Public Health (Hrvatski zavod za javno zdravstvo, [www.hzjz.hr](http://www.hzjz.hr)) | 1 | 1. Price (75)  2. The number of HPV genotypes contained in the vaccine (20)  3. Validity period at the time of delivery (5) | 31.5 |
| Croatia | **2016/S 040-065138,**  **EVV 10/15, EVV 47/15**  **(Source TED,** [**www.hzjz.hr**](http://www.hzjz.hr)**,** eojn.nn.hr **& Personal Communication)** | 18.08.2015  And  18.11.2015 | 2-valent  and  4-valent | 1,848,000  HRK (970,200 used finally)  and  3,347,867  HRK  (1 HRK = 0.131469675 EUR) | 255.577  and  440,143 | 4 months  and  1 year | - | 14,000 (7,000 used finally)  and  14.000 | 14,000  14,000 | Croatian Institute for Public Health (Hrvatski zavod za javno zdravstvo, [www.hzjz.hr](http://www.hzjz.hr)) | 2 | 1. Price (70)  2. Genotype (20)  3. Validity period (10) | 17.4  and  31.4 |
| Estonia | **2017/S 206-425226**  **(Source TED)** | 16.10.2017 | 9-valent | - | 1,647,528 | 2 years | - | 38,000 | 19,000 | Republic of Estonia Ministry of Social Affairs Sotsiaalministeerium ([www.sm.ee](http://www.sm.ee)) | 2 | 1. Price(33)  2. Quality and effectiveness (67) | 43.4 |
| Hungary-Budapest | **2014/S 098-171253**  **(Source TED,** [**www.antsz.hu**](http://www.antsz.hu)**)** | 01.04.2014 | 2-valent | 1,689,600,000 HUF  (1 HUF= 0.003227906EUR) | 5,453,870 | 3 years | 80,000 total | 160,000 (60,000, 50,000, 50,000) | 50,000 | National Public Health and Medical Officer Service (Országos Tisztifőorvosi Hivatal, [www.antsz.hu](http://www.antsz.hu)) | 2 | 1. Price (80)  2. Prevention of Genital Warts (20) | 34.1 |
| Hungary-Budapest Capital IV. district Újpest | [**www.ujpest.hu**](http://www.ujpest.hu) | 07.11.2012 | 4-valent | 15,100 HUF per dose | - | 1.5 year | 400 persons per year | 1,200 | 1,200 | Budapest Főváros IV. district Municipality of Újpest (Budapest Főváros IV. kerület Újpest ) ([**www.ujpest.hu**](http://www.ujpest.hu)**)** | 2 | 1. Price | 48.7 |
| Hungary-Budapest  (Capital XI. district Újbuda) | **2010/S 9-009962**  **(source TED,** [**www.ujbuda.hu**](http://www.ujbuda.hu)**)** | 31.12.2009 | 2-valent | 19,215 HUF per dose  In totlal: 80,703,,000 HUF  (1 HUF= 0.003227906EUR) | 260,502 | 1 year | 1,400  (persons) | 4,200 | 4,200 | Budapest Capital XI. district Újbuda (Budapest Főváros XI. kerület Újbuda Önkormányzata, [www.ujbuda.hu](http://www.ujbuda.hu)) | 2 | 1. Price | 62.02 |
| Hungary- Hódmezővásárhely | **2008/S 231-306595**  **(Source TED)** | 30.10.2008 | 2-valent | 69,480,000 HUF  (1 HUF= 0.003227906EUR) | 224,275 | 5 years | - | 3,000  (600 doses per year) | 600 | Hódmezővásárhely County Council (Hódmezővásárhely Megyei Jogú Város Önkormányzata, [www.hodmezovasarhely.hu](http://www.hodmezovasarhely.hu)) | 2 | 1. Net price (10)  2. Annual price indices measured by the KSH published last year's inflation (1)  3. Anti-HPV16 and anti-HPV18 seroconversion after 3 months (%) | 74.8 |
| Hungary-Kecskemét | **2013/S 195-336419**  **(Source TED)** | 09.06.2013 | 4-valent | 108,720,000 HUF  (1 HUF= 0.003227906EUR) | 350,938 | 4 years | 600 per year | 7,200 in total  (3 doses) | 1,800 | Kecskemét County Council (Kecskemét Megyei Jogú Város Önkormányzata, [www.kecskemet.hu](http://www.kecskemet.hu)) | 1 | 1. Price | 48.7 |
| Hungary-Kecskemét | [www.kecskemet.hu](http://www.kecskemet.hu) | 27.09.2012 | 4-valent | 45,300 HUF per vaccine series or 15,100 HUF per dose Total contract:  19, 026, 000 HUF (1 HUF= 0.003227906EUR) | 61,414 | 1 year | 420 series per year | Estimated 1,260 | 1,260 | Kecskemét County Council (Kecskemét Megyei Jogú Város Önkormányzata, [www.kecskemet.hu](http://www.kecskemet.hu)) | 1 | 1. Price | 48.7 |
| Hungary-Kecskemét | **2011/S 241-390288 (Source TED)** | 9**.**12.2011 | 4-valent | 12,324,750 HUF (1 HUF= 0.003227906EUR) | 39,783 | 1 | 250 series per year | 750 doses | 750 | Kecskemét County Council (Kecskemét Megyei Jogú Város Önkormányzata, [www.kecskemet.hu](http://www.kecskemet.hu)) | 1 | 1. Gross Order Price (10)  2. Component Number (2 or 4, Advantage of Multiple)(1) | 53.0 |
| Hungary-Kecskemét | **2010/S 159-245134 (Source TED,** [www.kecskemet.hu](http://www.kecskemet.hu)) | 06.09.2011 | 4-valent | 51,764 HUF per vaccine series (122.261.520 HUF) Total (1 HUF= 0.003227906EUR) | 394,649 | 1 | 620 series (+40% ) | 1,860 doses (+40% ) | 1,860 | Kecskemét County Council (Kecskemét Megyei Jogú Város Önkormányzata, [www.kecskemet.hu](http://www.kecskemet.hu)) | 2 | 1. Gross Order Price (10)  2. Component Number (2 or 4, Advantage of Multiple)(1) | 55.7 |
| Hungary-Százhalombatta | **2008/S 232-307954**  **(Source TED)** | 17.11.2008 | 4-valent | 42,450,800 HUF  (1 HUF= 0.003227906EUR) | 137.027 | 4 years |  | 1,800  (450 doses per year) | 450 | Százhalombatta City Council (Százhalombatta Város Önkormányzata, [www.szazhalombatta.hu](http://www.szazhalombatta.hu)) | 1 | 1. Price | 76.1 |
| Hungary-Százhalombatta | **2007/S 231-281101**  **(Source TED)** | 20.11.2007 | 4-valent | 57,214,916 HUF  (1 HUF= 0.003227906EUR) | 184,684 | 1 year | 674 | 2,022 | 2,022 | Százhalombatta City Council (Százhalombatta Város Önkormányzata, [www.szazhalombatta.hu](http://www.szazhalombatta.hu)) | 1 | 1. Price | 91.3 |
| Hungary-Szolnok | **2008/S 214-284718**  **(Source TED)** | 27.10.2008 | 4-valent | 105,998,628 HUF  (1 HUF= 0.003227906EUR) | 342153,61 | 4 years | 1,743 | 5,229 | 1,307 | Municipality of Szolnok County (Szolnok Megyei Jogú Város Önkormányzata, [www.info.szolnok.hu](http://www.info.szolnok.hu) ) | 2 | 1. Judicial considerations 2. The lowest amount of remuneration | 65.4 |
| Iceland^[[2]](#footnote-2)^ | **2017/S 074-144149**  **(Source TED,** [**www.rikiskaup.is**](http://www.rikiskaup.is)**)** | 29.12.2016 | 2-valent | 2,801 ISK per dose(1ISK= 0.006834959 EUR) | - | 2 years | - | 3,600 per year  (2000 * 0.9*2 doses) | 3,600 | Directorate of Health ([www.landlaeknir.is](http://www.landlaeknir.is)) | 2 | The most economically advantageous tender in terms of the criteria stated in the specifications, in the invitationto tender or to negotiate or in the descriptive document | 19.2 |
| Iceland | **2012/S 89-145994**  **(Source TED,** [**www.rikiskaup.is**](http://www.rikiskaup.is)**)** | 20.06.2011 | 2-valent | 3,596 ISK per dose(1ISK= 0.006834959 EUR) | - | 4 years | - | 5,400 per year  (2000 * 0.9* 3 doses) | 5,400 | Directorate of Health ([www.landlaeknir.is](http://www.landlaeknir.is)) | 2 | The most economically advantageous tender in terms of the criteria stated in the specifications, in the invitationto tender or to negotiate or in the descriptive document | 24.6 |
| Italy-Abruzzo | **2012/S 30-047540**  **(Source TED,** [**www.asl2abruzzo.it**](http://www.asl2abruzzo.it)**)** | 20.07.2011 | 4-valent | - | 3,308,760 | 3 years | - | 63,000  (21,000 per year) | 21,000 | Health Service of the Abruzzo Region (Azienda sanitaria locale 2 Lanciano Vasto Chieti, <http://www.asl2abruzzo.it>) | 2 | 1. Price | 52.5 |
| Italy-Emilia Romagna | [**intercenter.regione.emilia-romagna.it**](http://intercenter.regione.emilia-romagna.it) | 02.10.2017 | 9-valent | - | 2,154,537 | 1-year | - | 34,199 | 34,199 | Intercent-ER Regional Agency (Agenzia Regionale Intercent-ER, [intercenter.regione.emilia-romagna.it/agenzia](http://intercenter.regione.emilia-romagna.it/agenzia)) | 1 | 1. Price | 63.0 |
| Italy-Emilia Romagna | [**intercenter.regione.emilia-romagna.it**](http://intercenter.regione.emilia-romagna.it) | 15.05.2015 | 4-valent | - | 666,000 | 1,5-years | - | 20,000 | 20,000 | Intercent-ER Regional Agency (Agenzia Regionale Intercent-ER, [intercenter.regione.emilia-romagna.it/agenzia](http://intercenter.regione.emilia-romagna.it/agenzia)) | 1 | 1. Price | 33.3 |
| Italy-Emilia Romagna | **2013/S 046-073218 (Source TED,** [**intercenter.regione.emilia-romagna.it**](http://intercenter.regione.emilia-romagna.it)**)** | 01.04.2014  And  15.11.2012 | 4-valent |  | 8,101,800 | 3 years | - | 180,000 | 60,000 | Intercent-ER Regional Agency (Agenzia Regionale Intercent-ER, [intercenter.regione.emilia-romagna.it/agenzia](http://intercenter.regione.emilia-romagna.it/agenzia)) | 1  2 | 1. Price | 33.5  45.1 |
| Italy-Emilia Romagna | **2009/S 64-091325**  **(Source TED,** [**intercenter.regione.emilia-romagna.it**](http://intercenter.regione.emilia-romagna.it) **)** | 31.03.2009 | 2-valent | - | 9,125,000 | 3 years | - | 182,500 | 60,830 | Intercent-ER Regional Agency (Agenzia Regionale Intercent-ER, [intercenter.regione.emilia-romagna.it/agenzia](http://intercenter.regione.emilia-romagna.it/agenzia)) | 2 | 1. Technical offer (35)  2. Economic offer (65) | 50.0 |
| Italy-Emilia Romagna | **2008/S 65-087292 (Source TED,** [**intercenter.regione.emilia-romagna.it**](http://intercenter.regione.emilia-romagna.it)**)** | 06.03.2008 | 2-balent | - | 3,356,000 | 1 year | - | 40,000 | 40,000 | Intercent-ER Regional Agency (Agenzia Regionale Intercent-ER, [intercenter.regione.emilia-romagna.it/agenzia](http://intercenter.regione.emilia-romagna.it/agenzia)) | 2 | 1. Price | 83.9 |
| Italy-Friuli Venezia Gulia | **2016/S 050-082805**  **(Source TED,** [**www.egas.sanita.fvg.it**](http://www.egas.sanita.fvg.it)**)** | 29.02.2016^[[3]](#footnote-3)^ | 4-valent | - | 1,045,471  (501417, 544054.5) | 2 years | - | 30450  (14700 , 15950) | 15,225 | Regional Health Service of the Autonomous Region of Friuli Venice Giulia (Aziende del servizio sanitario regionale del Friuli Venezia Giulia, [**www.csc.sanita.fvg.it**](http://www.csc.sanita.fvg.it)**)** | 1  2 | 1. Price (65)  2. Quality (35) | 34.1 |
| Italy-Friuli Venezia Gulia | **2013/S 123-210819**  **(Source TED,** [**www.albopretorio.regione.fvg.it/ap/aoud**](http://www.albopretorio.regione.fvg.it/ap/aoud)**)** | 21.06.2013 | 4-valent | - | 1,103,296  (value per year) | 2 years | - | 25,670 | 12,835 | Regional Health Service of the Autonomous Region of Friuli Venice Giulia (Aziende del servizio sanitario regionale del Friuli Venezia Giulia, [**www.csc.sanita.fvg.it**](http://www.csc.sanita.fvg.it)**)** | 2 | 1. Price (65)  2. Quality (35) | 43.0 |
| Italy-Friuli Venezia Gulia | **2010/S 215-329503**  **(Source TED,** [**www.csc.sanita.fvg.it**](http://www.csc.sanita.fvg.it)**)** | 03.11.2010 | 4-valent | - | 1,534,390 | 1 year | - | 29,000 | 29,000 | Regional Health Service of the Autonomous Region of Friuli Venice Giulia (Aziende del servizio sanitario regionale del Friuli Venezia Giulia, [**www.csc.sanita.fvg.it**](http://www.csc.sanita.fvg.it)**)** | 2 | 1. Price | 52.9 |
| Italy-Friuli Venezia Gulia | **2009/S 102-146495**  **(Source TED,** [**www.csc.sanita.fvg.it**](http://www.csc.sanita.fvg.it)**)** | 15.05.2009 | 2-valent | - | 1,516,183 | 1 year | - | 28,140 | 28,140 | Regional Health Service of the Autonomous Region of Friuli Venice Giulia (Aziende del servizio sanitario regionale del Friuli Venezia Giulia, [**www.csc.sanita.fvg.it**](http://www.csc.sanita.fvg.it)**)** | 2 | 1. Price | 53.9 |
| Italy-Friuli Venezia Gulia | **2008/S 144-192770**  **(Source TED,** [**www.csc.sanita.fvg.it**](http://www.csc.sanita.fvg.it)**)** | 10.04.2008 | 2-valent | - | 1,404,876 | 1 year | - | 17,790 | 17,790 | Regional Health Service of the Autonomous Region of Friuli Venice Giulia (Aziende del servizio sanitario regionale del Friuli Venezia Giulia, [**www.csc.sanita.fvg.it**](http://www.csc.sanita.fvg.it)**)** | 2 | 1. Price | 79.0 |
| Italy-Friuli Venezia Gulia | [**www.csc.sanita.fvg.it**](http://www.csc.sanita.fvg.it) | 10.07.2007 | 4-valent | - | 27,360 | 4 months | - | 240 | 240 | Regional Health Service of the Autonomous Region of Friuli Venice Giulia (Aziende del servizio sanitario regionale del Friuli Venezia Giulia, [**www.csc.sanita.fvg.it**](http://www.csc.sanita.fvg.it)**)** | 1 | 1. Price | 114 |
| Italy-Liguria | **2017/S 135-276687**  **(Source TED)** | 15.06.2017 | 9-valent | - | 4,074,840 | 3 years | - | 64,480 | 21,560 | A.li.sa. Ligurian Healthcare Service (A.li.sa. Azienda Ligure Sanitaria, [www.acquistiliguria.it](http://www.acquistiliguria.it)) | 1 | 1. Price | 63.0 |
| Italy-Liguria | **2014/S 025-039271**  **(Source TED,** [**www.acquistiliguria.it**](http://www.acquistiliguria.it)**)** | 27.01.2014 | 2-valent | - | 2,582,100 | 3 years | - | 67,950 | 22,650 | Regional Health Service of Liguria(AA.SS.LL della Regione Liguria, [www.acquistiliguria.it](http://www.acquistiliguria.it)) | 2 | 1. Price | 38.0 |
| Italy-Liguria | **2010/S 136-208582**  **(Source TED,** [**www.acquistiliguria.it**](http://www.acquistiliguria.it)**)** | 31.05.2010 | 2-valent | - | 4.448.736 | 3 years | - | 86,400 | 28,800 | Regional Health Service of Liguria (Aziende sanitarie della regione Liguria, [www.acquistiliguria.it](http://www.acquistiliguria.it)) | 2 | 1. Price | 51.5 |
| Italy-Liguria | **2008/S 54-072652**  **(Source TED,** [**www.asl2.liguria.it**](http://www.asl2.liguria.it)**)** | 13.02.2008 | 2-valent | - | 4,852,400 | 1 year | - | 56,000 | 56,000 | The Savonese Local Health Authority (Azienda Sanitaria Locale n°2 "Savonese", [www.asl2.liguria.it](http://www.asl2.liguria.it)) | 2 | 1. Price | 86.7 |
| Italy-Lombardy | **ARCA_2016_7_9**  **(source** [**www.sintel.regione.lombardia.it**](http://www.sintel.regione.lombardia.it)**)** | 18.10.2016 | 4-valent | - | 3,109,750 | 1 year | - | 88,850 | 88,850 | Central Regional Purchasing Agency for Lombardy Region (Azienda Regionale Centrale Acquisti per la Regione Lombardia, [www.arca.regione.lombardia.it](http://www.arca.regione.lombardia.it)) | 2 | 1. Price | 32.5 |
| Italy-Lombardy | **ARCA_2015_12**  **(source** [**www.sintel.regione.lombardia.it**](http://www.sintel.regione.lombardia.it)**)** | 21.10.2015 | 4-valent | - | 3,052,695 | 1 year | - | 90,050 | 90,050 | Central Regional Purchasing Agency for Lombardy Region (Azienda Regionale Centrale Acquisti per la Regione Lombardia, [www.arca.regione.lombardia.it](http://www.arca.regione.lombardia.it)) | 2 | 1. Price | 33.9 |
| Italy-Lombardy | **2014/S 226-399198**  **ARCA_2014_03**  **(Source TED,** [**www.sintel.regione.lombardia.it**](http://www.sintel.regione.lombardia.it)) | 12.9.2014 | 2-valent | - | 2,725,500 | 1 year | - | 94,800 | 94,800 | Central Regional Purchasing Agency for Lombardy Region (Azienda Regionale Centrale Acquisti per la Regione Lombardia, [www.arca.regione.lombardia.it](http://www.arca.regione.lombardia.it)) | 2 | 1. Price | 28.8 |
| Italy-Lombardy | **2010/S 145-223017**  **(Source TED,** [**www.lispa.it**](http://www.lispa.it)**)** | 15.06.2010 | 2-valent | - | 8,945,200 | 1.5 years | - | 190,000 | 126,667 | Regional Health Bureaus  Lombardy (Enti Sanitari della Regione Lombardia, Lombardia Informatica S.p.A., [www.lispa.it](http://www.lispa.it)) | 2 | 1. Price | 47.1 |
| Italy-Lombardy | **2008/S 195-257986 (Source TED,** [**www.lispa.it**](http://www.lispa.it)**)** | 14.07.2008 | 4-valent | - | 8,523,992 | 1.5 years | - | 124,456 | 82,971 | Regional Health Bureaus  Lombardy (Enti Sanitari della Regione Lombardia, Lombardia Informatica S.p.A., [www.lispa.it](http://www.lispa.it)) | 2 | 1. Price | 68.5 |
| Italy-Marche^[[4]](#footnote-4)^ | **2015/S 153-282129**  **(Source TED,** [**www.asur.marche.it**](http://www.asur.marche.it)**)** | 06.11.2015 | 2-valent and  4-valent | - | 659,600 and  34,400 | 1 year | - | 19,400  and  1,000 | 19,400  And  1,000 | Regional Health Authority of Marche (Azienda Sanitaria Unica Regionale – ASUR, [www.asur.marche.it](http://www.asur.marche.it)) | 2 | 1. Price | 34.0  and  34.4 |
| Italy-Marche | **2013/S 249-435721**  **(Source TED,** [**www.asur.marche.it**](http://www.asur.marche.it)**)** | 18.10.2013 | 2-valent | - | 931,000 | 1 year^[[5]](#footnote-5)^ | - | 24,500 | 24,500 | Regional Health Authority of Marche (Azienda Sanitaria Unica Regionale – ASUR, [www.asur.marche.it](http://www.asur.marche.it)) | 2 | 1. Price | 38.0 |
| Italy-Marche | **2011/S 199-323660**  **(Source TED,** [**www.asur.marche.it**](http://www.asur.marche.it)**)** | 12.9.2011 | 2-valent | - | 1,230,000 | 1 year | - | 25,000 | 25,000 | Regional Health Authority of Marche (Azienda Sanitaria Unica Regionale – ASUR, [www.asur.marche.it](http://www.asur.marche.it)) | 2 | 1. Price | 49.2 |
| Italy-Piemonte | **2017/S 054-100443 (Source TED,** [**www.scr.piemonte.it**](http://www.scr.piemonte.it)**)** | 22.02.2017 | 2-valent | - | 2,658,500 | 2 years | - | 81,800 | 40,900 | Regional Health Service of Piemonte Region (Aziende  del Servizio Sanitario della Regione Piemonte, [www.scr.piemonte.it](http://www.scr.piemonte.it)) | 1 | 1. Price | 32.5 |
| Italy-Piemonte | **2013/S 123-210732 (Source TED,** [**www.scr.piemonte.it**](http://www.scr.piemonte.it)**)** | 20.06.2013 | 2-valent | - | 3,981,285 | 3 years | - | 115,500 | 38,500 | Regional Health Service of Piemonte Region (Aziende  del Servizio Sanitario della Regione Piemonte, [www.scr.piemonte.it](http://www.scr.piemonte.it)) | 2 | 1. Price | 34.5 |
| Italy-Piemonte | **2010/S 227-346504 (Source TED,** [**www.scr.piemonte.it**](http://www.scr.piemonte.it)**)** | 07.10.2010 | 4-valent | - | 9,368,000 | 2 years | - | 200,000 | 100,000 | Regional Health Service of Piemonte Region (Aziende  del Servizio Sanitario della Regione Piemonte, [www.scr.piemonte.it](http://www.scr.piemonte.it))) | 2 | 1.Price | 48.2 |
| Italy-Piemonte | **2009/S 183-262993**  **(Source TED)** | 07.09.2009 | 4-valent | - | 3,917,858 | 1 year | - | 84,255 | 84,255 | Regional Health Service of Piemonte Region (AA.SS.LL. della Regione Piemonte, [www.asl1.to.it](http://www.asl1.to.it) ) | 2 | 1. Price | 46.5 |
| Italy-Piemonte | **2008/S 217-288344 (Source TED)** | 17.10.2008 | 2-valent | - | 4,089,960 | 1 year | - | 64,920 | 64,920 | Regional Health Service of Piemonte Region (AA.SS.LL. della Regione Piemonte, [www.asl1.to.it](http://www.asl1.to.it) ) | 2 | 1. Price | 63.0 |
| Italy-Puglia | [**www.empulia.it**](http://www.empulia.it) | 26.09.2017 | 9-valent | - | 1,260,000+441,000 | 1 year | - | 27,000  (20,000 + 7,000) | 27,000 | REGIONE PUGLIA (  AZIENDA SANITARIA LOCALE DELLA PROVINCIA DI FOGGIA, REGIONE PUGLIA  AZIENDA SANITARIA LOCALE PROVINCIA DI BARI) | 1 | 1. Price | 63.0 |
| Italy-Puglia^[[6]](#footnote-6)^ | **2015/S 238-432239 (Source TED, www.empulia.it)** | 14.10.2016 | 4-valent | - | - | 2 years | - | 72,400 | 36,200 | Regional Health Agency of the Puglia region (Agenzia Regionale Sanitaria della Regione Puglia, [www.sanita.puglia.it](http://www.sanita.puglia.it) ) | 1 | 1. Price  2. Quality | 33.0 |
| Italy-Puglia | **2015/S 238-432239 (Source TED, www.empulia.it)** | 08.10.2015 | 2-valent | - | 7,893,600 | 3 years | - | 276,000 | 92,000 | Regional Health Agency of the Puglia region (Agenzia Regionale Sanitaria della Regione Puglia, [www.sanita.puglia.it](http://www.sanita.puglia.it) ) | 1 | 1. Price  2. Quality | 28.6 |
| Italy-Puglia | **2008/S 118-156420 (Source TED, www.arespuglia.it)** | 14.05.2008 | 2-valent | - | 4,666,230 | 1 year | - | 62,500 | 62,500 | Regional Health Agency of the Puglia region (Agenzia Regionale Sanitaria della Regione Puglia, [www.arespuglia.it](http://www.arespuglia.it) ) | 2 | 1. Value of supply (vaccine characteristics, packaging, dose and syringe presentation) (60)  2. Price (35)  3. Professional insertion (5) | 74.7 |
| Italy-Sicily | [**www.pti.regione.sicilia.it**](http://www.pti.regione.sicilia.it)**,** [**www.appalti.regione.sicilia.it**](http://www.appalti.regione.sicilia.it)**)** | 04.07.2017 | 9-valent | - | 9,150,750 | 1.5 years | - | 145,250 | 145,250 | Sicily's Central Commissariat (Centrale Unica di Committenza della Regione Siciliana, [www.pti.regione.sicilia.it](http://www.pti.regione.sicilia.it)) | 1 | 1. Price | 63.0 |
| Italy-Sicily | **2016/S 122-219059 (Source TED)** | 14.10.2015 | 4-valent | - | 8,360,000 | 3 years | - | 250,000 | 83,333 | Provincial Sanitary Company of Trapani (Azienda Sanitaria Provinciale di Trapani, [www.asptrapani.it](http://www.asptrapani.it) ) | 2 | 1. Price | 33.4 |
| Italy-Sicily | **2016/S 122-219059 (Source TED)** | 14.10.2015 | 2-valent | - | 2,454,500 | 3 years | - | 74,600 | 24,867 | Provincial Sanitary Company of Trapani (Azienda Sanitaria Provinciale di Trapani, [www.asptrapani.it](http://www.asptrapani.it) ) | 2 | 1. Price | 32.5 |
| Italy-Trento | **2016/S 231-420752 (Source TED)** | 28.10.2016 | 4-valent | - | 599,250 | 1 year | - | 15,000^[[7]](#footnote-7)^ | 15,000 | Health Services of Trento (Azienda Provinciale per i Servizi Sanitari (APSS) di TrentoVia, [www.apss.tn.it](http://www.apss.tn.it)) | 2 | 1. Price | 40.0 |
| Italy-Trento | **2014/S 153-274814 (Source TED,** [**www.apss.tn.it**](http://www.apss.tn.it)**)** | 04.08.2014 | 4-valent | - | 267,000 | 1 year | - | 6,000 | 6,000 | Provincial Health Services of Trento (Azienda provinciale servizi sanitari di Trento, [www.apss.tn.it](http://www.apss.tn.it)) | 2 | 1. Price | 44.5 |
| Italy-Trento | **2012/S 173-285495 (Source TED)** | 17.08.2012 | 2-valent | - | 371,280 | 2 years | - | 14,000  (7,000 per year) | 7,000 | Provincial Health Services of Trento (Azienda provinciale servizi sanitari di Trento, [www.apss.tn.it](http://www.apss.tn.it)) | 2 | 1. Price | 53.1 |
| Italy-Umbria | [**www.uslumbria1.gov.it**](http://www.uslumbria1.gov.it) | 24.10.2017 | 4-valent | - | 1,032,37.6 | 2 years | - | 30,004 | 15,002 | Regional Health Authority of Umbria (Umbria Salute S.c. a r.l. — Centrale Regionale per gli Acquisti in Sanità (CRAS), [www.uslumbria1.gov.it](http://www.uslumbria1.gov.it)) | 2 | 1. Price | 34.4 |
| Italy-Umbria | **2016/S 007-008117**  **(Source TED,** [**www.uslumbria1.gov.it**](http://www.uslumbria1.gov.it)**)** | 14.10.2015 | 4-valent | - | 660,480 | 2 years | - | 38,400 | 19,200 | Regional Health Authority of Umbria (Umbria Salute S.c. a r.l. — Centrale Regionale per gli Acquisti in Sanità (CRAS), [www.uslumbria1.gov.it](http://www.uslumbria1.gov.it)) | 2 | 1. Price | 34.4 |
| Italy-Umbria | **2013/S 217-377370 (Source TED, personal communication)** | 21.05.2013 | 4-valent | - | 1,242,338 | 2 years |  | 24,540 | 12,270 | Regional Health Authority of Umbria (Umbria Salute S.c. a r.l. — Centrale Regionale per gli Acquisti in Sanità (CRAS), [www.uslumbria1.gov.it](http://www.uslumbria1.gov.it)) | 2 | 1. Price | 45.0 |
| Italy-Umbria | **2009/S 243-347258**  **(Source TED, personal communication)** | 17.9.2009 | 2-valent | - | 2,173,892 | 2 years |  | 40,878 | 20,439 | Regional Health Authority of Umbria (Umbria Salute S.c. a r.l. — Centrale Regionale per gli Acquisti in Sanità (CRAS), [www.uslumbria1.gov.it](http://www.uslumbria1.gov.it)) | 2 | 1. Price | 62.7 |
| Italy-Umbria | **2008/S 153-205752**  **(Source TED, personal communication)** | 15.5.2008 | 2-valent | - | 1,772,382 | 1 year |  | 21,354 | 21,354 | Regional Health Authority of Umbria (Umbria Salute S.c. a r.l. — Centrale Regionale per gli Acquisti in Sanità (CRAS), [www.uslumbria1.gov.it](http://www.uslumbria1.gov.it)) | 2 | 1. Price | 83.0 |
| Italy-Veneto | [**www.regione.veneto.it**](http://www.regione.veneto.it) | 17.05.2017 | 9-valent | - | 16,650,900 | 3 years | - | 264,300 | 88,100 | Veneto Region - Regional Council (Regione del Veneto - Giunta Regionale, [www.regione.veneto.it](http://www.regione.veneto.it)) | 1 | 1. Price | 63.0 |
| Italy-Veneto | **2013/S 186-320858**  **(Source TED,** [**www.regione.veneto.it**](http://www.regione.veneto.it)**)** | 19.09.2013 | 4-valent | - | 8,568,218 | 3.5 years | - | 261,625 | 74,750 | Veneto Region - Regional Council (Regione del Veneto - Giunta Regionale, [www.regione.veneto.it](http://www.regione.veneto.it)) | 2 | 1. Price | 32.8 |
| Italy-Veneto | **2011/S 205-333336**  **(Source TED)** | 29.07.2011 | 4-valent | - | 7,320,015 | 2 years | - | 156,444 | 78,222 | Veneto Region - Regional Council (Regione del Veneto - Giunta Regionale, [www.regione.veneto.it](http://www.regione.veneto.it)) | 2 | 1. Price | 46.8 |
| Italy-Veneto | **2011/S 205-333336**  **(Source TED)** | 20.02.2009 | 4-valent | - | 12,542,970 | 2 years | - | 228,054 | 114,027 | Veneto Region - Regional Council (Regione del Veneto - Giunta Regionale, [www.regione.veneto.it](http://www.regione.veneto.it), [www.ulssvicenza.it](http://www.ulssvicenza.it) ) | 2 | 1. Price (50) 2. Quality (50) | 55.0 |
| Latvia | **2013/S 215-373986**  **(Source TED,** [www.vmnvd.gov.lv](http://www.vmnvd.gov.lv), personal communication**)** | 23.10.2013 | 2-valent | 619,183.50 LVL | 881,332(1 LVL = 1.422287 EUR) | 2 years | - | 36,725 | 18,363 | National Health Service (Nacionālais veselības dienests, [www.vmnvd.gov.lv](http://www.vmnvd.gov.lv)) | 2 | 1. Price | 24.0 |
| Latvia | **2011/S 234-378439 (Source TED,** [www.vmnvd.gov.lv](http://www.vmnvd.gov.lv), personal communication**)** | 25.11.2011 | 2-valent | 694 984,70 LVL | 988,468 (1 LVL = 1.422287 EUR) | 2 years |  | 39,110 | 19,555 | National Health Service (Nacionālais veselības dienests, [www.vmnvd.gov.lv](http://www.vmnvd.gov.lv)) | 2 | 1. Price | 25.3 |
| Latvia | **2009/S 235-335953**  **(Source TED)** | 25.11.2009 | 2-valent | 670,785.5 LVL | 953,857  (1 LVL = 1.422 EUR) | 2 years | - | 20,174 | 10,087 | National Health Service (Nacionālais veselības dienests, [www.vmnvd.gov.lv](http://www.vmnvd.gov.lv)) | 2 | 1. Price | 47.3 |
| Netherlands^[[8]](#footnote-8)^ | **2012/S 119-196951**  **CA-300-607**  **(Source TED,** [**www.nza.nl**](http://www.nza.nl)**)** | 01.01.2013 | 2-valent | - | - | 3 year  (1 year + 3) | 60,000 girls per year | 420,000 | 140,000 | National Institute of Public Health and the Environment (Rijksinstituut voor Volksgezondheid en Milieu ,www.rivm.nl) | 2 | 1. Price (60)  2. Scope of supply (25)  3. Delivery schedule (15) | 17.15 |
| Netherlands | **2012/S 119-196951**  **CA-300-567**  **(Source TED,** [**www.nza.nl**](http://www.nza.nl) **)** | 01.01.2012 | 2-valent | - | - | 1 year  (1 year + 3) | 60,000 girls per year | 180,000 | 180,000 | National Institute of Public Health and the Environment (Rijksinstituut voor Volksgezondheid en Milieu ,www.rivm.nl) | 2 | 1. Price (60)  2. Scope of supply (25)  3. Delivery schedule (15) | 17.15 |
| Norway | **2009/S 168-242116**  **(Source TED)** | 08.05.2009 | 4-valent | 170,000,000 NOK | 18,101,501  (1 NOK = 0.106479 EUR) | 2 year | - | 200,000 | 100,000 | Norwegian Institute of Public Health ([www.fhi.no](http://www.fhi.no)) | 2 | 1. Quality (70)  2. Price.(20)  3. Effect against condylomas (10) | 90.5 |
| Poland - Krakow | **2010/S 171-261416**  **ZP 3/2010**  **(source TED,** [**www.zeromski-szpital.pl**](http://www.zeromski-szpital.pl) **)** | 27.03.2010 | 2-valent | 31,030 PLN | 6,905  (1 PLN = 0,239285 EUR) | 1 year | - | 100 | 100 | Specialist Hospital Stefan Żeromski Independent Public Health Care Center in Kraków ([www.zeromski-szpital.pl](http://www.zeromski-szpital.pl) ) | 2 | 1. Price | 69.1 |
| Poland-Kwidzyn | **2015/S 166-303340**  **OR.IV.272.11.2015r**  **(Source Ted )** | 20.08.2015 | 4-valent | 978,750 PLN | 234,200  (1 PLN = 0,239285 EUR) | 1 year | - | 4,500 | 4,500 | County District Office in Kwidzyn (Starostwo Powiatowe w Kwidzynie, [www.bip.powiatkwidzynski.pl](http://www.bip.powiatkwidzynski.pl) ) | 1 | 1. Price (94)  2. Time to implement III dose vaccination (6) | 52.0 |
| Poland - Nowy Sacz | **2015/S 140-258210**  **DA.271-22/15**  **(source TED,** [**www.szpitalnowysacz.pl**](http://www.szpitalnowysacz.pl) **)** | 30.06.2015 | 4-valent | 251,910 PLN | 60,278  (1 PLN = 0,239285 EUR) | 1 year | - | 3,000 | 3,000 | Specialist Hospital Jędrzej Śniadecki (Szpital Specjalistyczny im. Jędrzeja Śniadeckiego, [www.szpitalnowysacz.pl](http://www.szpitalnowysacz.pl) ) | 2 | 1. Price (90)  2. Deadline for the current order (10) | 20.1 |
| Poland-Olsztyn | **2017/S 048-087949**  **EDZ.242.152/16**  **(Source Ted)** | 09.02.2017 | 2-valent | 2,948.72 PLN | 706  (1 PLN = 0,239285 EUR) | 1 year | - | 30 | 30 | Independent Public Clinical Hospital No. 4 in Lublin (Samodzielny Publiczny Szpital Kliniczny nr 4 w Lublinie, [www.spsk4.lublin.pl](http://www.spsk4.lublin.pl) ) | 1 | 1 .Price | 23.5 |
| Poland- Poznan | **2016/S 041-067219**  **EA-ZP.272.12.2015**  **(source TED)** | 15.12.2015 | 4-valent | 3 888,57 PLN | 930  (1 PLN = 0,239285 EUR) | 1 year | - | 21 | 21 | Provincial Sanitary-Epidemiological Station in Poznań (Wojewódzka Stacja Sanitarno-Epidemiologiczna w Poznaniu, [www.wsse-poznan.pl](http://www.wsse-poznan.pl) ) | 2 | 1. Price (99)  2. The delivery period for individual lots of vaccines (1) | 44.3 |
| Poland- Poznan | **2014/S 027-042824**  **EA-ZP.272.15.2013**  **(source TED)** | 09.12.2013 | 4-valent | 5,044.7 PLN | 1,303  (1 PLN = 0,239285 EUR) | 1 year | - | 30 | 30 | Provincial Sanitary-Epidemiological Station in Poznań (Wojewódzka Stacja Sanitarno-Epidemiologiczna w Poznaniu, [www.wsse-poznan.pl](http://www.wsse-poznan.pl) ) | 2 | Price | 43.4 |
| Poland- Poznan | **2013/S 014-018060**  **EA-ZP.272.17.2012**  **(Source TED)** | 21.11.2012 | 4-valent | 3,888.99 PLN | 931  (1 PLN = 0,239285 EUR) | 1 year | - | 21 | 21 | Provincial Sanitary-Epidemiological Station in Poznań (Wojewódzka Stacja Sanitarno-Epidemiologiczna w Poznaniu, [www.wsse-poznan.pl](http://www.wsse-poznan.pl) ) | 2 | Price | 44.3 |
| Poland-Szczecin | **2015/S 030-050803**  **1/2015**  **(Source TED,** [**www.zamowienia.szpital-zdroje.pl**](http://www.zamowienia.szpital-zdroje.pl) **)** | 03.02.2015 | 4-valent | 1,048,249.50 PLN | 250,830.4  (1 PLN = 0,239285 EUR) | 1 year | 1500 girls | 4,500 | 4,500 | Independent Public Specialist Health Care Center "Zdroje" | 1 | 1. Price (95)  2. Time to implement one vaccination course. (5) | 55.7 |
| Poland-Wrocław | **WCZ/P/D -7 /2017**  [**www.spzoz.wroc.pl**](http://www.spzoz.wroc.pl) | 03.08.2017 | 4-valent | 313,187.47 PLN | 74,941  (1 PLN = 0,239285 EUR) | 1 year | 1730 girls | 3,460 | 3,460 | Wrocławskie Centrum Zdrowia SP ZOZ ([**www.spzoz.wroc.pl**](http://www.spzoz.wroc.pl)**)** | 1 | Price (90)  Payment deadline for the invoice (10) | 21.7 |
| Poland-Wrocław | **WCZ / P / D -7/2016**  [**www.spzoz.wroc.pl**](http://www.spzoz.wroc.pl) | 08.08.2016 | 4-valent | 374,654.85 PLN | 89,649.3  (1 PLN = 0,239285 EUR) | 1 year | 2074 girls | 4,148 | 4,148 | Wrocławskie Centrum Zdrowia SP ZOZ ([**www.spzoz.wroc.pl**](http://www.spzoz.wroc.pl)**)** | 2 | Price (90)  Payment deadline for the invoice (10) | 21.6 |
| Poland-Wrocław | **WCZ/P/D -5/2015**  [**www.spzoz.wroc.pl**](http://www.spzoz.wroc.pl) | 08.07.2015 | 4-valent | 369824,04 PLN | 88,493.3  (1 PLN = 0,239285 EUR) | 1 year | 2029 girls | 4,058 | 4,058 | Wrocławskie Centrum Zdrowia SP ZOZ ([**www.spzoz.wroc.pl**](http://www.spzoz.wroc.pl)**)** | 2 | Price (60)  Number of serotypes in the vaccine (40) | 21.8 |
| Poland-Wrocław | **2010/S 131-200023**  **PN/ZP/D- 1**  **(Source Ted,** [**www.spzoz.wroc.pl**](http://www.spzoz.wroc.pl)**)** | 05.07.2010 | 2-valent | 1,119,810 PLN | 267,953.7  (1 PLN = 0,239285 EUR) | 1 year | 1900 girls^[[9]](#footnote-9)^ | 5,700 | 5,700 | Wrocławskie Centrum Zdrowia SP ZOZ ([**www.spzoz.wroc.pl**](http://www.spzoz.wroc.pl)**)** | 2 | Lowest price  The most economically advantageous tender in terms of criteria | 47.0 |
| Portugal**^[[10]](#footnote-10)^** | **Source contracts published on**  [**www.base.gov.pt**](http://www.base.gov.pt) | 29.12.2016 | 9-valent | 1,610,000  650,000  110,000  129,520 | - | 1 year | - | 73,800 | 73,800 | - Administração Regional de Saúde de Lisboa e Vale do Tejo - Administração Regional de Saúde do Algarve, IP - Unidade de Saúde da Ilha do Faial | 1 | - | 40.0 |
| Portugal | **2015/S 245-444879**  **Source TED**  **Source contracts published on**  [**www.base.gov.pt**](http://www.base.gov.pt) | 24.04.2014 | 4-valent | - | - | 2 years | - | 147,600 | 73,800 | - Administração Regional de Saúde do Norte, I. P. - Administração Regional de Saúde de Lisboa e Vale do Tejo, I. P. - Unidade Local de Saúde de Castelo Branco, E. P. E. | 2 | - | 22.0 |
| Portugal | **2014/S 127-226694**  **CP 2013/17 HPV**  **Source TED**  **Source contracts published on**  [**www.base.gov.pt**](http://www.base.gov.pt) | 14.05.2013 | 4-valent | - | - | 2 years | - | 221,400 | 110,700 | - Administração Regional de Saúde de Lisboa e Vale do Tejo, IP - Unidade de Saúde da Ilha de São Miguel - Administração Regional de Saúde do Alentejo, I. P. - Unidade Local de Saúde de Castelo Branco, E. P.E | 2 | - | 29.9 |
| Portugal | **2008/S 210-278630**  **Source TED**  **cp 2008/16**  **Source contracts published on**  [**www.base.gov.pt**](http://www.base.gov.pt) | 17.10.2008 | 4-valent | 9,303,470 | - | 2 years | - | 227,550 | 113,775 | Administração Regional de Saúde de Lisboa e Vale do Tejo, IP, Administração Regional de Saúde do Norte, I.P, | 2 | - | 41.0 |
| Portugal^[[11]](#footnote-11)^ | **2009/S 94-134288**  **2008017/438/0002**  **CP 2008/17**  **(Source TED)** | 08.08.2008 | 4-valent | - | - | 1 year | - | 110,700 | 110,700 | Central Administration of the Health System (Administração Central do Sistema de Saúde IP, [www.acss.min-saude.pt](http://www.acss.min-saude.pt) ) | 2 | 1. Comparative evaluation of vaccines (57)  2. Price (43) | 57.8 |
| Slovenia | **2017/S 013-019501**  **2016/S 227-413312**  **16K150415**  **(Source TED, personal communication)** | 16.06.2016 | 9-valent |  |  | 3 years |  | 35,000 | 11,667 | National Institute of Public Health (Nacionalni inštitut za javno zdravje, [www.nijz.si/sl](http://www.nijz.si/sl)) | 1 | The most economically advantageous tender in terms of the criteria stated in the specifications, the invitation to tender or the start of the negotiations and the information package | 49.0 |
| Slovenia | **Source: Personal communication** | 18.08.2013 | 4-valent | - | - | 3 years |  | 31,956 | 10,652 | National Institute of Public Health (Nacionalni inštitut za javno zdravje, [www.nijz.si/sl](http://www.nijz.si/sl)) | 2 | The most economically advantageous tender in terms of the criteria stated in the specifications, the invitation to tender or the start of the negotiations and the information package | 35.85 |
| Slovenia | **Source: Personal communication** | 15.10.2012 | 4-valent | - | - | 1 year |  | 17,584 | 17,584 | National Institute of Public Health (Nacionalni inštitut za javno zdravje, [www.nijz.si/sl](http://www.nijz.si/sl)) | 2 | The most economically advantageous tender in terms of the criteria stated in the specifications, the invitation to tender or the start of the negotiations and the information package | 37.73 |
| Slovenia | **2011/S 71-115627**  **7/11 JN, 4300-002/11/024**  **(Source: TED, personal communication)** | 19.06.2012 | 4-valent |  |  | 1 year |  | 4,974 | 4,974 | National Institute of Public Health (nacionalni Institut za javno zdravje, [www.ivz-rs.si](http://www.ivz-rs.si) ) | 2 | The most economically advantageous tender in terms of the criteria stated in the specifications, the invitation to tender or the start of the negotiations and the information package | 55.0 |
| Slovenia | **2007/S 74-089789**  **900-037-005/07**  **(Source TED, personal communication)** | 18.06.2009 | 4-valent | - | - | 3 years | - | 40,336 | 13,445 | National Institute of Public Health ([www.ivz-rs.si](http://www.ivz-rs.si) ) | 2 | 1. Price | 70.0 |
| Spain-Andalusia | **Contract 2208/2010**  [**www.juntadeandalucia.es**](http://www.juntadeandalucia.es) | 20.05.2015 | 4-valent | 11.664.000 | - | 3 years  (30 months) | - | 400,000 | 133,333 | Andalucian Health Service (Servicio Andaluz de Salud, <http://www.juntadeandalucia.es> ) | 2 | 1.Economic offer  2.1. Genotype of the vaccine  2.2 technical characteristics  2.3 Complementary offers for the development of the Andalusian vaccination program or the improvement of its endowment and logistics | 29.16 |
| Spain-Andalusia | **2012/S 79-129344**  **CCA. +K1K8CK (2011/103758)**  **(Source Ted)** | 05.08.2011 | 2-valent | 15.624.000 | - | 2 years  (22 months) | - | 240,000 | 120,000^[[12]](#footnote-12)^ | Andalucian Health Service (Servicio Andaluz de Salud, <http://www.juntadeandalucia.es> ) | 2 | 1.Economic offer  2.1. Genotype of the vaccine  2.2 technical characteristics  2.3 Complementary offers for the development of the Andalusian vaccination program or the improvement of its endowment and logistics | 65.1 |
| Spain-Andalusia | **2009/S 134-195543**  **CCA. +QYLS2D**  **(Source Ted,** [**www.juntadeandalucia.es**](http://www.juntadeandalucia.es)**)** | 11.02.2009 | 2-valent | 11.634.595 | - | 1 year | - | 125,000 | 125,000^[[13]](#footnote-13)^ | Andalucian Health Service (Servicio Andaluz de Salud, <http://www.juntadeandalucia.es> ) | 2 | 1.Economic offer  2.1. Genotype of the vaccine  2.2 technical characteristics  2.3 Complementary offers for the development of the Andalusian vaccination program or the improvement of its endowment and logistics | 87.8 |
| Spain-Aragon | **SGT/29/2015**  **(**[**www.acobur.es**](http://www.acobur.es) **)** | 13.07.2015 | 2-valent | 272,459 | - | 1 year | - | 9,800  (450 gratis) | 9,800 | Department of Health and Consumption of the Government of Aragon Departamento de Salud y Consumo del Gobierno de Aragón, [**www.acobur.es**](http://www.acobur.es) **)** | 2 | 1. The most economically advantageous  2. Administratively advantageous | 29.1 |
| Spain-Aragon | **SGT/17/2013 (**[**www.acobur.es**](http://www.acobur.es) **)** | 14.10.2013 | 2-valent | 190.076 | - | 1 year | - | 6,100 | 6,100 | Department of Health and Consumption of the Government of Aragon Departamento de Salud y Consumo del Gobierno de Aragón, [**www.acobur.es**](http://www.acobur.es) **)** | 2 | 1. The most economically advantageous  2. Administratively advantageous | 31.2 |
| Spain-Aragon | **2012/S 112-185121**  **SGT/29/2012**  **(Source TED,** [**www.acobur.es**](http://www.acobur.es)**)** | 12.06.2012 | 4-valent | 1,372,500 | - | 1 year | - | 22,500 | 22,500 | Department of Health and Consumption of the Government of Aragon Departamento de Salud y Consumo del Gobierno de Aragón, [**www.acobur.es**](http://www.acobur.es) **)** | 2 | The economically most advantageous offer, taking into account administrative (100) | 61.0 |
| Spain-Aragon | **2011/S 194-315428**  **SGT/35/2011**  **(Source TED,** [**www.acobur.es**](http://www.acobur.es)**)** | 22.08.2011 | 4-valent | 872,300 | - | 1 year | - | 13,000 | 13,000 | Department of Health and Consumption of the Government of Aragon Departamento de Salud y Consumo del Gobierno de Aragón, [**www.acobur.es**](http://www.acobur.es) **)** | 2 | 1. Criteria dependent on value judgment(23)  2. Quantifiable criteria through formulas (74) | 67.1 |
| Spain-Aragon | **2010/S 210-320361**  **SGT/34/2010**  **(Source TED,** [**www.acobur.es**](http://www.acobur.es)**)** | 22.10.2010 | 4-valent | 943,200 | - | 1 year | - | 13,100 | 13,100 | Department of Health and Consumption of the Government of Aragon Departamento de Salud y Consumo del Gobierno de Aragón, [**www.acobur.es**](http://www.acobur.es) **)** | 2 | 1. Efficacy, effectiveness and immunogenicity(24)  2. Compatibility with other immunogens(1)  3. Price (50)  4. Technical characteristics vaccine (15)  5. Free extra doses given (10) | 72.0 |
| Spain-Aragon | **2009/S 233-332733 SGT/48/2009**  **(Source TED,** [**www.acobur.es**](http://www.acobur.es)**)** | 12.11.2009 | 4-valent | 1,499,850 | - | 1 year | - | 16,500 | 16,500 | Department of Health and Consumption of the Government of Aragon Departamento de Salud y Consumo del Gobierno de Aragón, [**www.acobur.es**](http://www.acobur.es) **)** | 2 | 1. Oncogenic types(16)  2. Oncogenic types not contained in vaccine (4)  3. Non-oncogenic viruses (2)  4. Compatibility with other immunogens (1)  5. Price (50)  6. Storage characteristics (4)  7. Improvements in the application (3)  8. Thermostability (3)  9. Expiration (3)  10. Free doses (2) | 90.9 |
| Spain-Asturias | **SC/71-14**  **(**[**www.sede.asturias.es**](http://www.sede.asturias.es) **)** | 12.12.2014 | 2-valent | 46745,2 | - | 1 year | - | 1,500 | 1,500 | Health Service of the Principality of Asturias (Servicio de Salud del Principado de Asturias, [**www.asturias.es**](http://www.asturias.es) **)** | 2 | 1. Price | 31.2 |
| Spain-Asturias | **SC/05-13**  **(**[**www.sede.asturias.es**](http://www.sede.asturias.es) **)** | 04.02.2013 | 2-valent | 249,280 | - | 1 year | - | 8,000 | 8,000 | Health Service of the Principality of Asturias (Servicio de Salud del Principado de Asturias, [**www.asturias.es**](http://www.asturias.es) **)** | 2 | 1. Price | 31.2 |
| Spain-Asturias | **SC/36-09 (**[**www.sede.asturias.es**](http://www.sede.asturias.es) **)** | 11.03.2010 | 4-valent | - | - | 1 year | - | 7,000 | 7,000 | Health Service of the Principality of Asturias (Servicio de Salud del Principado de Asturias, [**www.asturias.es**](http://www.asturias.es) **)** | 2 | 1. Economic offer (80)  2. Technical conditions (20) | 90.0 |
| Spain-Asturias | **2008/S 229-304003**  **6000/19-08 (**[**www.sede.asturias.es**](http://www.sede.asturias.es) **)** | 08.10.2008 | 4-valent | 1,948,500 | - | 2 years | - | 19,485  (5,485+14,000) | 9,743 | Health Service of the Principality of Asturias (Servicio de Salud del Principado de Asturias, [**www.asturias.es**](http://www.asturias.es) **)** | 1 | 1. Price and bonuses (100) | 100.0 |
| Spain-Govern Illes Balears | **DES01 2015 1791**  **(**[www.plataformadecontractacio.caib.es](http://www.plataformadecontractacio.caib.es)**)** | 11.05.2015 | 2-valent | 524,880x2 | - | 2 years |  | 36,000 | 18,000 | Government of the Balearic Islands, Ministry of Health (Govern de Illes Balears, Conselleria de Salut, [www.caib.es](http://www.caib.es)) | 2 | 1. Price (75)  2. Presentation that facilitates the application of the vaccine (10)  3. Additional or no-charge doses (5)  4. Replacement of expired vaccines (5)  5. Training (5) | 29.16 |
| Spain-Govern Illes Balears | **2014/S 129-230695**  **CONTR 2014 957**  **(Source TED,** [www.plataformadecontractacio.caib.es](http://www.plataformadecontractacio.caib.es)**)** | 05.06.2014 | 2-valent | 373,920 | - | 1 year |  | 12,000 | 12,000 | Government of the Balearic Islands, Ministry of Health (Govern de Illes Balears, Conselleria de Salut, [www.caib.es](http://www.caib.es)) | 1 | 1. Price  2. Technical specificities of the vaccine | 31.16 |
| Spain-Govern Illes Balears | **2012/S 209-343249**  **CONTR 2012 2646**  **(Source TED,** [www.plataformadecontractacio.caib.es](http://www.plataformadecontractacio.caib.es)**)** | 25.09.2012 | 2-valent | 1,394,230.77 | - | 2 years |  | 25,000  (10,000 and 15,000) | 12,500 | Government of the Balearic Islands, Ministry of Health (Govern de Illes Balears, Conselleria de Salut, [www.caib.es](http://www.caib.es)) | 2 | 1. Price (70)  2. Operational Improvement (10)  3.Best Techniques (20) | 47.9 |
| Spain-Govern Illes Balears | **2008/S 223-296424**  **SSCC CM 231/08**  **(Source TED,** [www.boe.es/boe/dias/2008/08/16/pdfs/B09894-09894.pdf](http://www.boe.es/boe/dias/2008/08/16/pdfs/B09894-09894.pdf)**)** | 23.09.2008 | 2-valent | 1,401,750 | - | 1 year |  | 17,500 | 17,500 | Health Service of the Balearic Islands (Servicio de Salud de las Illes Balears) | 2 | 1. Price (50)  2. Operational Improvement (10)  3.Technical Improvement (40) | 80.1 |
| Spain-Basque | **9/2018-S**  **(**[**www.contratacion.euskadi.eus**](http://www.contratacion.euskadi.eus) **)** | 27.12.2017 | 4-valent | 233,600  (max budget) | - | 1 year | - | 8,055 | 8,055  (max) | Basque Government, Department of Health  (Eusko Jaurlaritza-Gobierno Vasco, Departamento de Salud, [www.euskadi.eus/gobierno-vasco/departamento-salud](http://www.euskadi.eus/gobierno-vasco/departamento-salud)) | 2 | 1. Price (60)  2. Presentation (15)  3. Additional Antigens (10)  4. Bar code (5)  5. Delivery (5)  6. Expiration (5) | 29.2 |
| Spain-Basque | **2016/S 035-056258**  **C03/002/2015**  **(Source: Ted,** [**www.contratacion.euskadi.eus**](http://www.contratacion.euskadi.eus) **)** | 04.01.2016 | 4-valent | 742,500 | - | 1 year | - | 27,500 | 27,500 | Basque Government, Department of Health  (Eusko Jaurlaritza-Gobierno Vasco, Departamento de Salud, [www.euskadi.eus/gobierno-vasco/departamento-salud](http://www.euskadi.eus/gobierno-vasco/departamento-salud)) | 2 | 1. Price (65)  2. Presentation of the vaccines (15)  3. Additional Antigens (5)  4. Bar code (5)  5. Delivery (5)  6. Expiration (5) | 27.0 |
| Spain-Basque | **2014/S 046-076907**  **C03/003/2013**  **(Source: Ted,** [**www.contratacion.euskadi.eus**](http://www.contratacion.euskadi.eus) **)** | 16.01.2014 | 4-valent | 940,500 | - | 1 year | - | 28,500 | 28,500 | Basque Government, Department of Health  (Eusko Jaurlaritza-Gobierno Vasco, Departamento de Salud, [www.euskadi.eus/gobierno-vasco/departamento-salud](http://www.euskadi.eus/gobierno-vasco/departamento-salud)) | 2 | 1. Price (65)  2. Presentation of the vaccines (15)  3. Additional Antigens (5)  4. Bar code (5)  5. Delivery (5)  6. Expiration (5) | 31.3 |
| Spain-Basque | **TED 2012/S 166-274838 C03/002/12**  **(Source: Ted,** [**www.contratacion.euskadi.eus**](http://www.contratacion.euskadi.eus) **)** | 24.07.2012 | 4-valent | 1,722,500 | - | 1 year | - | 26,500 | 26,500 | Basque Government, Department of Health  (Eusko Jaurlaritza-Gobierno Vasco, Departamento de Salud, [www.euskadi.eus/gobierno-vasco/departamento-salud](http://www.euskadi.eus/gobierno-vasco/departamento-salud)) | 2 | 1. Price (60)  2. Additional vaccine doses without cost (10)  3. Additional antigens (10)  4. Presentation (5)  5. Barcode (5)  6. Term of expiration (5)  7. Improved delivery time (5) | 52.06 |
| Spain-Basque | **2009/S 126-182891 C03/004/2009**  **(Source: Ted**, [**www.contratacion.euskadi.eus**](http://www.contratacion.euskadi.eus) **)** | 26.07.2009 | 4-valent | 2,700,000 | - | 2 years | - | 30,000 per year | 30,000 | Basque Government, Department of Health  (Eusko Jaurlaritza-Gobierno Vasco, Departamento de Salud, [www.euskadi.eus/gobierno-vasco/departamento-salud](http://www.euskadi.eus/gobierno-vasco/departamento-salud)) | 1 | 1. Price (65)  2. Presentation of the vaccines (15)  3. Additional Antigens (5)  4. Bar code (5)  5. Delivery (5)  6. Expiration (5) | 90.0 |
| Spain-Basque | **2008/S 176-233936**  **C03/003/2008**  **(Source: Ted**, [**www.contratacion.euskadi.eus**](http://www.contratacion.euskadi.eus) **)** | 03.09.2008 | 4-valent | 2,700,000 | - | 1 year | - | 30,000 | 30,000 | Basque Government, Department of Health  (Eusko Jaurlaritza-Gobierno Vasco, Departamento de Salud, [www.euskadi.eus/gobierno-vasco/departamento-salud](http://www.euskadi.eus/gobierno-vasco/departamento-salud)) | 1 | 1. Price | 90.0 |
| Spain-Basque | **2007/S 211-255863 C03/007/2007 (www.irekia.euskadi.eus )** | 26.10.2007 | 4-valent | 3,000,000 | - | 1 year | - | 30,000 | 30,000 | Basque Government, Department of Health (Gobierno Vasco. Comisión Central de Contratación, [www.euskadi.eus/gobierno-vasco/departamento-salud](http://www.euskadi.eus/gobierno-vasco/departamento-salud)) | 1 | 1. Specific conditions of the vaccine  2. Delivery time | 100.0 |
| Spain-Cantabria | **2012/S 57-092359**  **P.A. 17/11**  **(Source: Ted)** | 16.02.2012 | 2-valent | 487,550 | - | 1 year | - | 7,000 | 7,000 | Cantabrian Health Service (Servicio Cántabro de Salud, [www.scsalud.es](http://www.scsalud.es)) | 2 | 1. Economic offer (5)  2. additional doses without charge (5)  3. presentation in campaign packaging (1)  4. storage possibility without cold network (1) | 69.7 |
| Spain-Cantabria | **2011/S 101-164937**  **P.A. 2/11**  **(Source: Ted)** | 16.05.2011 | 2-valent | 288,680 | - | 1 year | - | 4,000 | 4,000 | Cantabrian Health Service (Servicio Cántabro de Salud, [www.scsalud.es](http://www.scsalud.es)) | 2 | 1. price (5)  2. additional doses without charge (5)  3. best presentation in campaign packaging (1) | 72.2 |
| Spain-Cantabria | **2009/S 165-238511**  **P.A. 17/09**  **(Source: Ted)** | 30.07.2009 | 2-valent | 542,308 | - | 1 year  (6 months) | - | 6,000 | 6,000 | Cantabrian Health Service (Servicio Cántabro de Salud, [www.scsalud.es](http://www.scsalud.es)) | 2 | 1. price (5)  2. additional doses without charge (5)  3. best presentation in campaign packaging (1) | 90.4 |
| Spain-Cantabria | **2008/S 16-019496**  **C.A. 39/07**  **(Source: Ted)** | 18.01.2008 | 2-valent | 616,846 | - | 1 year | - | 6,480 | 6,480 | Cantabrian Health Service (Servicio Cántabro de Salud, [www.scsalud.es](http://www.scsalud.es)) | 2 | 1. economic proposal (5)  2. technical proposal (1)  3. complementary services (1) | 95.2 |
| Spain-Castile and Leon | **2012/S 97-161008**  **6/2012**  **(Source: Ted,** [**www.bocyl.jcyl.es/boletin.do?fechaBoletin=28/05/2012**](http://www.bocyl.jcyl.es/boletin.do?fechaBoletin=28/05/2012) **)** | 09.04.2012 | 4-valent | 1,945,800 | - | 1 year | - | 40,000 | 40,000 | COMMUNITY OF CASTILLA Y LEÓN, HEALTH COUNSELING  (COMUNIDAD DE CASTILLA Y LEÓN, CONSEJERÍA DE SANIDAD, [www.jcyl.es/](http://www.jcyl.es/) ) | 1 | 1. Price (40)  2. Presentation (10)  3. Composition (1)  4. Thermostability (1) | 48.6 |
| Spain-Castile and Leon | **2011/S 82-134282**  **09/2011**  **(Source: Ted)** | 31.03.2011 | 4-valent | 3,000,000 | - | 1 year | - | 40,000 | 40,000 | COMMUNITY OF CASTILLA Y LEÓN, HEALTH COUNSELING  (COMUNIDAD DE CASTILLA Y LEÓN, CONSEJERÍA DE SANIDAD, [www.jcyl.es/](http://www.jcyl.es/) ) | 2 | 1. Price (40)  2. Collaborations (5)  3. Additional advantages (12) | 75.0 |
| Spain-Castile and Leon | **2009/S 229-328402**  **23/09**  **(Source: Ted)** | 15.06.2009 | 4-valent | 1,890,000 | - | 1 year | - | 21,000 | 21,000 | COMMUNITY OF CASTILLA Y LEÓN, HEALTH COUNSELING  (COMUNIDAD DE CASTILLA Y LEÓN, CONSEJERÍA DE SANIDAD, [www.jcyl.es/](http://www.jcyl.es/) ) | 2 | 1. price (30)  2. advantages in the presentation (18)  3. collaborations (4) | 90.0 |
| Spain-Catalonia | **SA-2017-867 (**[**www.contractaciopublica.gencat.cat**](http://www.contractaciopublica.gencat.cat)**)** | 13.11.2017 | 9-valent | 3,306,000 | - | 2 years |  | 87,000 | 43,500 | Department of Health of the Government of Catalonia (Departament de Salut de la Generalitat de Catalunya, [www.salutweb.gencat.cat/ca/inici/](http://www.salutweb.gencat.cat/ca/inici/)) | 1 | Price. (Due to the object of the contract, no negotiation criteria are established, since it is only  can entrust a single successful bidder) | 38.0 |
| Spain-Catalonia | **2016/S 243-443285**  **SA-2016-577**  **(Source: Ted ,** [**www.contractaciopublica.gencat.cat**](http://www.contractaciopublica.gencat.cat)**)** | 08.11.2016 | 4-valent | 2,305,500 | - | 2 years | - | 87,000 | 43,500 | Department of Health of the Government of Catalonia (Departament de Salut de la Generalitat de Catalunya, [www.salutweb.gencat.cat/ca/inici/](http://www.salutweb.gencat.cat/ca/inici/)) | 2 | 1. Price (80)  2. Presentation that facilitates storage(3)  3. Security system (1)  4. Thermostability (2.5)  5. Expiration dates (2)  6. Vaccine characteristics (7)  7. other improvements (4.5) | 26.5 |
| Spain-Catalonia | **2015/S 219-399467**  **SA-2015-803**  **(Source: Ted ,** [**www.contractaciopublica.gencat.cat**](http://www.contractaciopublica.gencat.cat)**)** | 15.09.2015 | 4-valent | 1,869,840 | - | 2 years |  | 72,000 | 36,000 | Department of Health of the Government of Catalonia (Departament de Salut de la Generalitat de Catalunya, [www.salutweb.gencat.cat/ca/inici/](http://www.salutweb.gencat.cat/ca/inici/)) | 2 | 1. Price (80)  2. Presentation that facilitates storage (3)  3. Security system (1)  4. Thermostability (3)  5. Expiration dates (2)  6. Vaccine characteristics (7)  7. other improvements (4) | 26.0 |
| Spain-Catalonia | **2014/S 207-366578**  **SA-2014-570**  **(Source: Ted)** | 22.09.2014 | 4-valent | 2,098,000 | - | 2 years |  | 72,000 | 36,000 | Department of Health of the Government of Catalonia (Departament de Salut de la Generalitat de Catalunya, [www.salutweb.gencat.cat/ca/inici/](http://www.salutweb.gencat.cat/ca/inici/)) | 2 | 1. Price (80)  2. Presentation that facilitates storage (3)  3. Security system (1)  4. Thermostability (3)  5. Expiration dates (2)  6. Vaccine characteristics (7)  7. other improvements (4) | 29.1 |
| Spain-Catalonia | **154/2013**  **(Source:** [**www.acobur.es**](http://www.acobur.es)**,** [**www.elperfildelcontratante.es**](http://www.elperfildelcontratante.es) **)** | 10.04.2013 | 4-valent | 3,271,800 | - | 2 years |  | 105,000 | 52,500 | Department of Health of the Government of Catalonia (Departament de Salut de la Generalitat de Catalunya, [www.salutweb.gencat.cat/ca/inici/](http://www.salutweb.gencat.cat/ca/inici/)) | 2 | 1. Price (80)  2. Presentation that facilitates storage (3)  3. Presentation that facilitates the application of the vaccine (3)  4. Thermostability (3)  5. Expiration dates (3)  6. Vaccine characteristics (4)  7. other improvements (4) | 31.2 |
| Spain-Catalonia^[[14]](#footnote-14)^ | **123/2012**  **(Source:** [**www.acobur.es**](http://www.acobur.es)**,** [**www.elperfildelcontratante.es**](http://www.elperfildelcontratante.es) **)** | 30.07.2012 | 4-valent | 1,764,962 | - | 1 year |  | 53,000 | 53,000 | Department of Health of the Government of Catalonia (Departament de Salut de la Generalitat de Catalunya, [www.salutweb.gencat.cat/ca/inici/](http://www.salutweb.gencat.cat/ca/inici/)) | 2 | 1. Price (80)  2. Presentation that facilitates storage (3)  3. Security system (1)  4. Thermostability (3)  5. Expiration dates (2)  6. Vaccine characteristics (7)  7. other improvements (4) | 33.3 |
| Spain-Catalonia | **2012/S 121-200214**  **SA-2011-198**  **(Source: Ted)** | 09.09.2011 | 4-valent | 3,008,000 | - | 2 years |  | 84,000 | 42,000 | Department of Health of the Government of Catalonia (Departament de Salut de la Generalitat de Catalunya, [www.salutweb.gencat.cat/ca/inici/](http://www.salutweb.gencat.cat/ca/inici/)) | 2 | 1. Economic offer (80)  2. Technical assessment (20) | 35.8 |
| Spain-Catalonia | **2010/S 233-355868**  **180/2010**  **(Source: Ted)** | 04.10.2010 | 2-valent | 4,939,200 | - | 2 years |  | 105,000 | 52,500 | Department of Health of the Government of Catalonia (Departament de Salut de la Generalitat de Catalunya, [www.salutweb.gencat.cat/ca/inici/](http://www.salutweb.gencat.cat/ca/inici/)) | 2 | 1. Economic offer (80)  2. Technical characteristics (20) | 47.0 |
| Spain-Catalonia | **2009/S 14-018919**  **177/2008**  **(Source: Ted)** | 10.11.2008 | 4-valent | 16,379,956 | - | 2 years |  | 210,000 | 105,000 | Department of Health of the Government of Catalonia (Departament de Salut de la Generalitat de Catalunya, [www.salutweb.gencat.cat/ca/inici/](http://www.salutweb.gencat.cat/ca/inici/)) | 2 | 1. Economic offer (80)  2. Technical characteristics (20) | 78.0 |
| Spain-Ceuta | **2012/S 159-264898**  **100/11**  **(Source: Ted,** [**www.ceuta.es**](http://www.ceuta.es) **)** | 06.06.2012 | 2-valent | 165,000 | - | 2 years |  | 3,000 | 1,500 | Autonomous City of Ceuta (Ciudad Autonoma de Ceuta, [www.ceuta.es](http://www.ceuta.es)) | 1 | 1. economic offer (50)  2. better technical characteristics of the vaccine (30)  3. better vaccination logistics (15)  4. Objective improvements on the offer submitted (5) | 55.0 |
| Spain-Ceuta | **24872/11 or 32/11**  **(**[**www.ceuta.es**](http://www.ceuta.es) **)** | 18.11.2011 | 4-valent | 114,995  (119,595) | - | 1 year |  | 1,700 | 1,700 | Autonomous City of Ceuta (Ciudad Autonoma de Ceuta, [www.ceuta.es](http://www.ceuta.es)) | 2 | 1. economic offer (50)  2. technical characteristics of the vaccine (38)  3. Quality in the provision of the service (8)  4. Objective improvements on the offer submitted (4) | 67.6 |
| Spain-Ceuta | **2010/S 143-219609**  **137/09**  **(Source: Ted,** [**www.ceuta.es**](http://www.ceuta.es) **)** | 07.07.2010 | 4-valent | 161,500 | - | 1 year |  | 1,700 | 1,700 | Autonomous City of Ceuta (Ciudad Autonoma de Ceuta, [www.ceuta.es](http://www.ceuta.es)) | 2 | 1. economic offer (50)  2. technical characteristics of the vaccine (40)  3. quality in the presentation of the service (10) | 95.0 |
| Spain-Extremadura | **2012/S 102-169654**  **CS/99/1111056148/11/PA (Source: Ted)** | 13.04.2012 | 4-valent | 528,024 | - | 1 year |  | 9,800 | 9,800 | Health Service of Extremadura  (Servicio Extremeño de Salud, www.saludextremadura.ses.es/web/ ) | 2 | 1. Economic offer (80)  2. Qualities of vaccines and the implementation of the vaccination program (20) | 53.9 |
| Spain-Extremadura | **2011/S 122-20163**  **CS/99/1111007341/11/PA (Source: Ted)** | 10.06.2011 | 4-valent | 830,400 | - | 1 year |  | 12,000 | 12,000 | Health Service of Extremadura  (Servicio Extremeño de Salud, www.saludextremadura.ses.es/web/ ) | 2 | The economically most advantageous offer taking into account: the criteria that appear in the bid specifications, in the invitation to bid or negotiate or in the descriptive document | 69.2 |
| Spain-Extremadura | **2010/S 165-253060**  **CS/99/111006568/10/PA (Source: Ted)** | 12.07.2010 | 4-valent | 1,539,000 | - | 1 year |  | 17,100 | 17,100 | Health Service of Extremadura  (Servicio Extremeño de Salud, www.saludextremadura.ses.es/web/ ) | 2 | 1. economic offer (40)  2. Vaccine qualities and execution (60) | 90.0 |
| Spain-Extremadura | **2009/S 209-299872**  **CS/99/1109027439/09/PA (Source: Ted)** | 05.10.2009 | 4-valent | 1,485,000 | - | 1 year | - | 16,500 | 16,500 | Health Service of Extremadura  (Servicio Extremeño de Salud, www.saludextremadura.ses.es/web/ ) | 2 | 1. price (40)  2. qualities of the vaccine (40)  3. antigenic composition of the HPV vaccine (10) | 90.0 |
| Spain-Extremadura | **2008/S 198-261238**  **CS/99/1108033540/08/PA (Source: Ted)** | 30.09.2008 | 4-valent | 1,350,000 | - | 1 year | - | 15,000 | 15,000 | Health Service of Extremadura  (Servicio Extremeño de Salud, www.saludextremadura.ses.es/web/ ) | 2 | 1. Technical offer (50)  2. Economic offer (25) | 90.0 |
| Spain-Galicia | **NS-CON1-17-009 (**[**www.contratosdegalicia.gal**](http://www.contratosdegalicia.gal) **)** | 23.08.2017 | 2-valent | 370,332 | - | 1 year | - | 12,700 | 12,700 | Galician health service  (Servizo Galego de Saude, [www.sergas.es](http://www.sergas.es) ) | 2 | 1. Price (40)  2.Presentation that facilitates application  3. Thermostability  4. Presence of self-adhesive labels  5. Vaccine composition  6. Other improvements | 29.16 |
| Spain-Galicia | **NS-CON1-14-009**  **(**[**www.contratosdegalicia.gal**](http://www.contratosdegalicia.gal) **)** | 11.04.2014 | 2-valent | 482,980 | - | 1 year | - | 15,500 | 15,500 | Galician health service  (Servizo Galego de Saude, [www.sergas.es](http://www.sergas.es) ) | 2 | Price and other, although not explicitly mentioned | 31.16 |
| Spain-Galicia | **NS-CON1-13-003 (**[**www.contratosdegalicia.gal**](http://www.contratosdegalicia.gal) **)** | 07.05.2013 | 2-valent | 118,644 | - | 2 years | - | 35,900 | 17,950 | Galician health service  (Servizo Galego de Saude, [www.sergas.es](http://www.sergas.es) ) | 2 | Price and other, although not explicitly mentioned | 31.16 |
| Spain-Galicia | **2012/S 233-382554**  **MS-CON1-12-005**  **(Source Ted,** [**www.contratosdegalicia.gal**](http://www.contratosdegalicia.gal)**)** | 21.09.2012 | 4-valent | 467,460 | - | 1 year | - | 8,820 | 8,820 | Galician health service  (Servizo Galego de Saude, [www.sergas.es](http://www.sergas.es) ) | 2 | 1. Economic offer (50)  2. Contributions (3)  3. Thermostability characteristics (4)  4. Expiration characteristics (3)  5. Presentation improvements that facilitate the application (5)  6. Presentation improvements that facilitate storage (2)  7. Improvements that facilitate logistics and the information system (1) 8. Protective efficacy against other types of oncogenic HPV (3)  9. Protective efficacy against other types of non-oncogenic HPV (1) | 53.0 |
| Spain-Galicia^[[15]](#footnote-15)^ | **2011/S 50-081410**  **MS-CON1-10-038**  **(Source: Ted,** [**www.contratosdegalicia.gal**](http://www.contratosdegalicia.gal) **)** | 12.03.2011 | 2-valent | 2,272,725 | - | 2 years | - | 35,000 | 17,500 | Galician health service  (Servizo Galego de Saude, [www.sergas.es](http://www.sergas.es) ) | 2 | Price | 64.9 |
| Spain-Madrid | **Reference**  **1992265 (**[**www.madrid.org**](http://www.madrid.org)**)** | 21.12.2017 | 2-valent | 4,968,280.8 | - | 3  years | - | 170.380 | 56,793 | Community of Madrid, Madrilenian Health Service (Comunidad de Madrid, Servicio Madrileno de Salud, ) | 2 | 1. price  2. free replenishment of dose after incident in the cold chain  3. time at which the vaccine is stable at room temperature  4. Vaccine composition (antigens) | 29.2 |
| Spain-Madrid | **2012/AC002**  **Reference**  **264128**  **(**[**www.madrid.org**](http://www.madrid.org)**)** | 23.12.2014 | 2-valent | 779,000 | - | 1 year | - | 25,000 | 25,000 | Community of Madrid, Madrilenian Health Service (Comunidad de Madrid, Servicio Madrileno de Salud, [www.madrid.org](http://www.madrid.org)) | 2 | Price and other | 31.2 |
| Spain-Madrid | **2012/AC002**  **Reference**  **255800**  **(**[**www.madrid.org**](http://www.madrid.org)**)** | 19.02.2014 | 2-valent | 2,492,800 | - | 1 year | - | 80,000 | 80,000 | Community of Madrid, Madrilenian Health Service (Comunidad de Madrid, Servicio Madrileno de Salud, [www.madrid.org](http://www.madrid.org) ) | 2 | Price and other | 31.2 |
| Spain-Madrid | **2012/AC002**  **Reference**  **248377**  **(**[**www.madrid.org**](http://www.madrid.org)**)** | 08.05.2013 | 2-valent | 1,589,160 | - | 1 year | - | 51,000 | 51,000 | Community of Madrid, Madrilenian Health Service (Comunidad de Madrid, Servicio Madrileno de Salud, [www.madrid.org](http://www.madrid.org) ) | 2 | Price and other | 31.2 |
| Spain-Madrid | **2012/S 43-069308**  **PA 11/2011**  **(Source TED)** | 19.12.2011 | 2-valent | 4,435,535 | - | 1 year | - | 83,700 | 83,700 | Community of Madrid, Madrilenian Health Service (Comunidad de Madrid, Servicio Madrileno de Salud, [www.madrid.org](http://www.madrid.org) ) | 2 | Price | 53.0 |
| Spain-Madrid | **2010/S 234-357690**  **2012/AC002**  **203755, P.A. 18/2010 (Source Ted,** [**www.madrid.org**](http://www.madrid.org)**)** | 22.02.2011 | 4-valent | 6,000,000 | - | 1 year | - | 80,000 | 80,000 | Community of Madrid, Madrilenian Health Service (Comunidad de Madrid, Servicio Madrileno de Salud, [www.madrid.org](http://www.madrid.org) ) | 2 | 1. Price (70)  2. Technical Improvements (30) | 75.0 |
| Spain-Madrid | **File number**  **P.A. 04/10**  **Reference**  **188816**  **(**[**www.madrid.org**](http://www.madrid.org)**)** | 29.06.2010 | 4-valent | 4,320,000 | - | 1 year | - | 48,000 | 48,000 | Community of Madrid, Madrilenian Health Service (Comunidad de Madrid, Servicio Madrileno de Salud, [www.madrid.org](http://www.madrid.org) ) | 2 | Price and other | 90.0 |
| Spain-Madrid | **2009/S 139-202596**  **Reference**  **166332**  **P.A. 05/08-TA**  **(Source: TED,** [**www.madrid.org**](http://www.madrid.org)**)** | 10.07.2009 | 4-valent | 4,050,000 | - | 1 year | - | 45,000 | 45,000 | Community of Madrid, Madrilenian Health Service (Comunidad de Madrid, Servicio Madrileno de Salud, [www.madrid.org](http://www.madrid.org) ) | 2 | 1. Economic offer (60)  2. Improvement of technical characteristics (40) | 90.0 |
| Spain-Madrid | **2008/S 105-140465**  **Reference**  **SUM-44/2007.10-PAC (Source: Ted,** [**www.madrid.org**](http://www.madrid.org)**)** | 22.05.2008 | 4-valent | 8,886,028 | - | 1 year | - | 89,723 | 89,723 | Institute of Public Health (Instituto de Salud Pública, [www.madrid.org](http://www.madrid.org) ) | 2 | 1. Price (60)  2. Improvements (40) | 99.0 |
| Spain-Castilla-La Mancha | **2012/S 245-402282**  **SC 01/13**  **(Source TED,** [**www.appeconomia3.jccm.es**](http://www.appeconomia3.jccm.es) **)** | 17.11.2012 | 4-valent | 144,240 | - | 1 year | - | 3,000 | 3,000 | Junta de Comunidades de Board of Communities of Castilla-La Mancha. Department of Health and Social Affairs (Castilla-La Mancha. Consejería de Sanidad y Asuntos Sociales, [www.castillalamancha.es](http://www.castillalamancha.es) ) | 2 | 1. Price (80)  2. Technical proposal (10)  3. Complementary benefits (10) | 48.08 |
| Spain-Castilla-La Mancha | **2012/S 115-189736**  **SC 16/12**  **(Source TED,** [**www.appeconomia3.jccm.es**](http://www.appeconomia3.jccm.es) **)** | 15.06.2012 | 4-valent | 280,000 | - | 1 year | - | 5,000 | 5,000 | Junta de Comunidades de Board of Communities of Castilla-La Mancha. Department of Health and Social Affairs (Castilla-La Mancha. Consejería de Sanidad y Asuntos Sociales, [www.castillalamancha.es](http://www.castillalamancha.es) ) | 2 | 1. Price (80)  2. Technical proposal (5)  3. Supplementary services (15) | 56.0 |
| Spain-Castilla-La Mancha | **2011/S 31-050525**  **SC 6/2011**  **(Source TED,** [**www.pagina.jccm.es**](http://www.pagina.jccm.es)**)** | 07.02.2011 | 4-valent | 1,600,000 | - | 1 year | - | 25,000 | 25,000 | Junta de Comunidades de Board of Communities of Castilla-La Mancha. Department of Health and Social Affairs (Castilla-La Mancha. Consejería de Sanidad y Asuntos Sociales, [www.castillalamancha.es](http://www.castillalamancha.es) ) | 2 | 1. Price (50)  2. Improvements (35)  3. Technical characteristics of vaccines (15) | 64.0 |
| Spain-Castilla-La Mancha | **2010/S 30-042277**  **SC 05/10**  **(Source TED,** [**www.pagina.jccm.es**](http://www.pagina.jccm.es)**)** | 22.12.2009 | 4-valent | 2,970,000 | - | 1 year | - | 33,000 | 33,000 | Junta de Comunidades de Board of Communities of Castilla-La Mancha. Department of Health and Social Affairs (Castilla-La Mancha. Consejería de Sanidad y Asuntos Sociales, [www.castillalamancha.es](http://www.castillalamancha.es) ) | 2 | 1. Price (50)  2. Improvements (35)  3. Technical characteristics of vaccines (15) | 90.0 |
| Spain-Castilla-La Mancha | **2010/S 30-042277**  **9/2008**  **(Source TED,** [**www.pagina.jccm.es**](http://www.pagina.jccm.es)**)** | 25.01.2008 | 4-valent | 3,569,280 | - | 1 year | - | 33,000 | 33,000 | Junta de Comunidades de Board of Communities of Castilla-La Mancha. Department of Health and Social Affairs (Castilla-La Mancha. Consejería de Sanidad y Asuntos Sociales, [www.castillalamancha.es](http://www.castillalamancha.es) ) | 1 | Negotiated without a call for tenders | 108.2 |
| Spain-Melilla | **2009/S 196-280998**  **Vauna/2009**  **(Source TED,** [**www.melilla.es**](http://www.pagina.jccm.es)**)** | 01.09.2009 | 2-valent | 142,217.55 | - | 1 year | - | 1,590 | 1,590 | Autonomous City of Melilla, Ministry of Contracting and Heritage (Ciudada Autónoma de Melilla, Consejería de Contratación y Patrimonion, [www.melilla.es](http://www.melilla.es) ) | 2 | 1. Compatibility study (30)  2. Presentation (5)  3. Logistical support for transport and maintenance of the cold chain (10)  4. Collaboration (15)  5. Economic offer (40) | 89.5 |
| Spain-Melilla | **2008/S 107-142745**  **ML/Vacunas 2008**  **(Source TED,** [**wwww.melilla.es**](http://www.pagina.jccm.es)**)** | 13.05.2008 | 2-valent | 148,500 | - | 1 year | - | 1,500 | 1,500 | Autonomous City of Melilla, Ministry of Contracting and Heritage (Ciudada Autónoma de Melilla, Consejería de Contratación y Patrimonion, [www.melilla.es](http://www.melilla.es) ) | 2 | 1. Improvements offered (60)  2. Best price offered (40) | 99.0 |
| Spain-Murcia | **8/2017 (L-16) (**[**www.carm.es**](http://www.carm.es) **)** | 07.04.2017 | 2-valent | 641,080 | - | 3 years | - | 22,000 | 7,300 | Autonomous Community of the Region of Murcia (Comunidad Autónoma de la Región de Murcia, [**www.carm.es**](http://www.carm.es)**)** | 2 | 1. unit price (40)  2. characteristics of the vaccine (36)  3. No charge (18)  4. Storage (2)  4. ease of application (2)  5. barcode (2) | 29.14 |
| Spain-Murcia | **13/2015**  **(**[**www.carm.es**](http://www.carm.es)**)** | 10.06.2015 | 2-valent | 349,680 | - | 2 years | - | 12,000 | 6,000 | Autonomous Community of the Region of Murcia (Comunidad Autónoma de la Región de Murcia, [**www.carm.es**](http://www.carm.es)**)** | 2 | 1. unit price (50)  2. characteristics of the vaccine (35)  3. Storage (3)  4. ease of application (3)  5. barcode (5) | 29.14 |
| Spain-Murcia | **08/2013**  **(**[**www.carm.es**](http://www.carm.es) **)** | 11.03.2013 | 2-valent | 1,558,000 | - | 2 years | - | 50,000 | 25,000 | Autonomous Community of the Region of Murcia (Comunidad Autónoma de la Región de Murcia, [**www.carm.es**](http://www.carm.es)**)** | 2 | 1. unit price (50)  2. characteristics of the vaccine (32)  3. No charge dosis (3)  4. Storage (3)  3. ease of application (3)  5. barcode (5)  6. training hours (4) | 31.16 |
| Spain-Murcia | **2012/S 87-142262**  **2/2012**  **(Source: TED)** | 07.03.2012 | 4-valent | 2,040,500 | - | 1 year | - | 40,000 | 40,000 | Autonomous Community of the Region of Murcia (Consejería de Sanidad y Política Social de la Comunidad Autónoma de la Región de MurciaRonda, [**www.carm.es**](http://www.carm.es)**)** | 2 | 1. Economic offer (5)  2. Presentation of preparations (1)  3. Improvements in the application of vaccine preprarado and improvements in the storage facility (1) | 51.0 |
| Spain-Murcia | **2011/S 92-149912**  **3/2011**  **(Source: TED,** [**www.carm.e**](http://www.carm.e) **)** | 09.03.2011 | 2-valent | 1,320,000 | - | 1 year | - | 22,000 | 22,000 | Department of Health and Social Policy of the Autonomous Community of the Region of Murcia (Comunidad Autónoma de la Región de Murcia, [**www.carm.es**](http://www.carm.es)**)** | 2 | 1. Economic offer (4)  2. Presentation of preparations (1)  3. Improvements in the application of vaccine preparation (1) | 60.0 |
| Spain-Murcia | **2010/S 100-150764**  **1/2010**  **(Source: TED)** | 31.03.2010 | 2-valent | 940,500 | - | 1 year | - | 11,000 | 11,000 | Department of Health and Social Policy of the Autonomous Community of the Region of Murcia (Comunidad Autónoma de la Región de Murcia, [**www.carm.es**](http://www.carm.es)**)** | 2 | 1. Economic offer (5)  2. Presentation of preparations (1)  3. Improvements in the application of the vaccine preparation (1) | 85.5 |
| Spain-Murcia | **2009/S 78-111510**  **3/2009**  **(Source: TED)** | 20.03.2009 | 2-valent | 2,060,856 | - | 1 year | - | 25,200 | 25,200 | Department of Health and Social Policy of the Autonomous Community of the Region of Murcia (Comunidad Autónoma de la Región de Murcia, [**www.carm.es**](http://www.carm.es)**)** | 2 | 1. price (5)  2. label with barcode (1)  3. stability of the preparation (0.75)  4. improvements in storage (0.75)  5. improvements of the vaccine preparation (0.75) | 81.8 |
| Spain-Murcia | **2008/S 74-099441**  **1/2008**  **(Source: TED)** | 22.02.2008 | 2-valent | 2,494,800 | - | 1 year | - | 25,200 | 25,200 | Department of Health and Social Policy of the Autonomous Community of the Region of Murcia (Comunidad Autónoma de la Región de Murcia, [**www.carm.es**](http://www.carm.es)**)** | 2 | 1. price (5)  2. label with barcode (1)  3. stability of the preparation (0.75)  4. improvements in storage (0.75)  5. improvements of the vaccine preparation (0.75) | 99.0 |
| Sweden | **2015/S 005-005208**  **(Source TED,** [**www.sklkommentus.se**](http://www.sklkommentus.se)**)** | 15.12.2014 | 4-valent | - | - | 3- years | 55,000 per year | 320,000 | 110,000 | SKL Kommentus Purchasing Center (SKL Kommentus Inköpscentral , [www.sklkommentus.se](http://www.sklkommentus.se)) | 2 | 1. Price  2. Medical and pharmaceutical efficacy | 150.6 SEK per dose  Or  15.8 Euros (1 SEK = 0.10483 EUR) |
| Sweden | **LS 0910-0855**  **(Source** [**www.konkurrensverket.se**](http://www.konkurrensverket.se) **)** | 05.03.2010 | 2-vaelnt | 35,850,000 SEK per year | 3,758,156  Euros (1 SEK = 0.10483 EUR) | 3 years | 55,000 per year | 450,000 | 150,000 | SKL Kommentus Purchasing Center (SKL Kommentus Inköpscentral , [www.sklkommentus.se](http://www.sklkommentus.se)) | 2 | 1.Effect on cervical cancer (type specific effects on CIN2+ CIN3+)  2. Vaccine effect against progenitor cervical cancer effects  3. Effect on the constitutions need to be made after vaccination  4.Vaccine effects on other cancers  5. Side-effects and safety data  6. Assessment of storage and transport | 25.0 |

**Figure 1. Tender-based per-dose prices of the HPV vaccines stratified per country or region versus the date of the contract award**


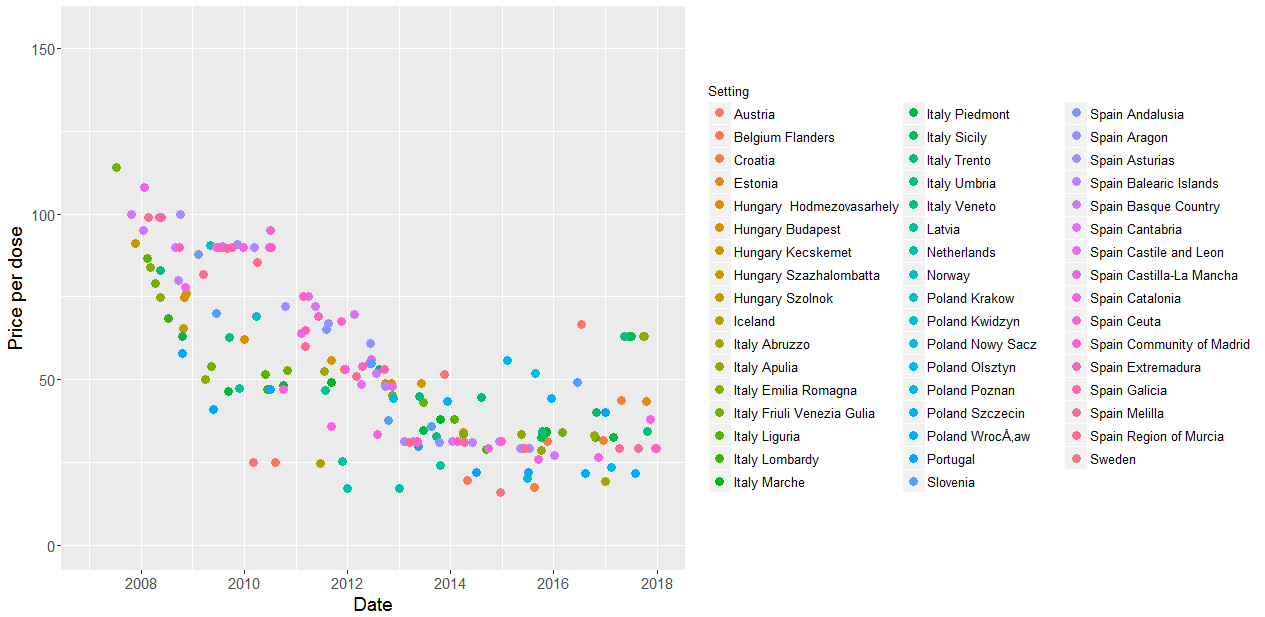


**Figure 2. Contract volume per vaccine type versus contract award date**

**
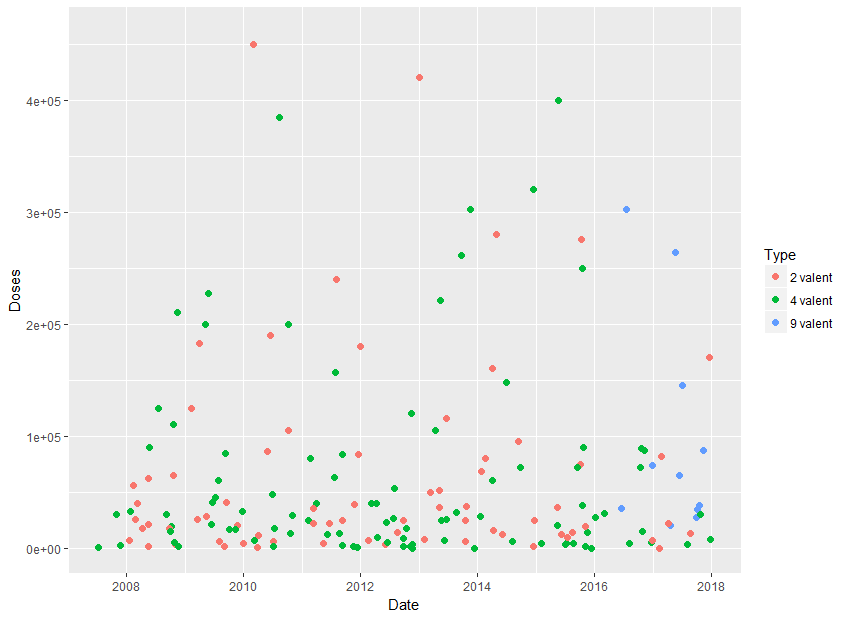
**

**Figure 3. Contract volume/per year versus contract award date for the three HPV vaccines**

**
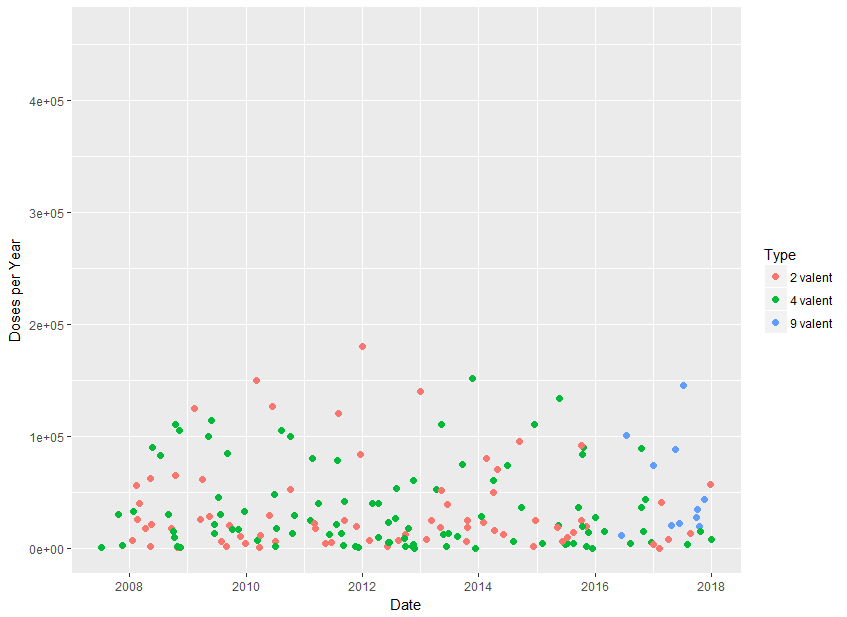
**

**Figure 4. Contract volume/per year versus contract award date for the different settings**

**
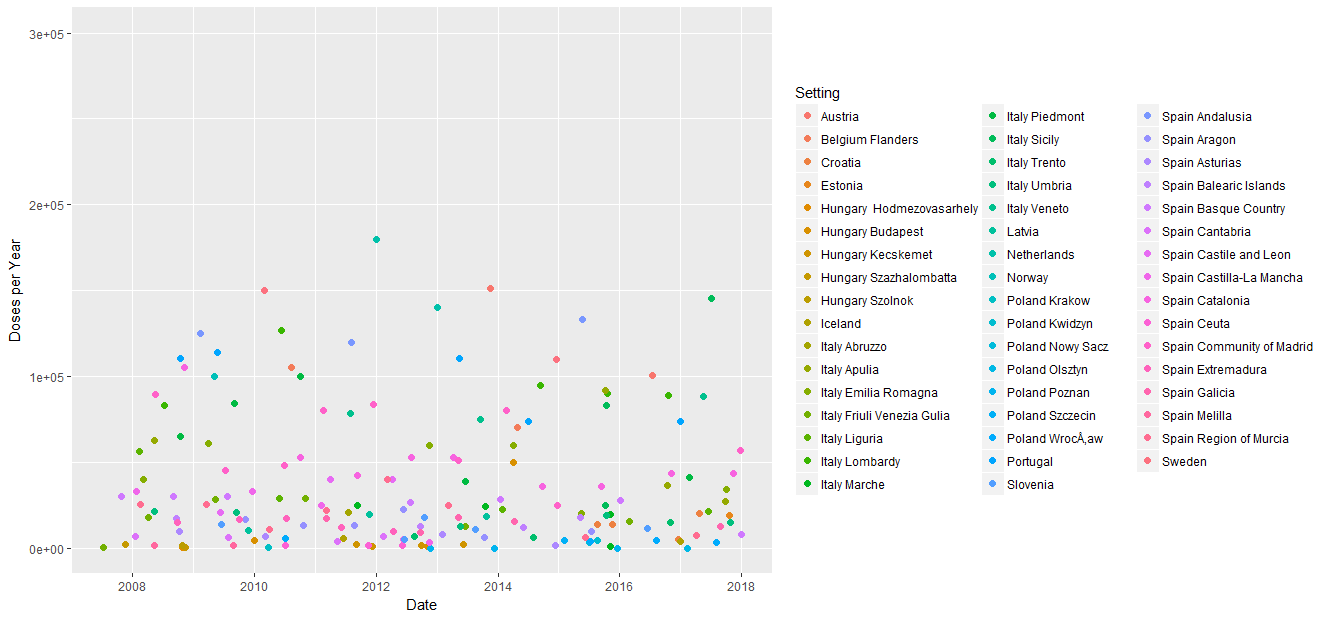
**

**Table 2. Estimates of different linear mixed models of tender-based prices for HPV vaccines**

| **Fixed effects** | **Model with random intercept only**  **(Model 1)** | **Model with random intercept and a natural cubic spline for time with df=2**  **(Model 2)** | **Model with random intercept, a natural cubic spline for time (df=2) and vaccine type**  **(Model 3)** | **Multiple linear mixed model with random intercept and a natural cubic spline for time (df=2)**  **(Model 4)** | |
| --- | --- | --- | --- | --- | --- |
| **Intercept** | 51.9** | 100.2** | 92.3** | 78.7** | |
| (SE) | (2.3) | (3.03) | (2.72) | (7.89) | |
| **ns(days)1** |  | -113.5** | -112.65** | -108.4** | |
| (SE) |  | (5.9) | (4.6) | (4.4) | |
| **ns(days)2** |  | -25.6** | -38.11** | -38.2** | |
| (SE) |  | (3.08) | (2.79) | (2.9) | |
| **Vaccine Type:** |  |  |  |  | |
| **4-valent** |  |  | 9.9** | 7.5** | |
| (SE) |  |  | (1.64) | (1.6) | |
| **9-Valent** |  |  | 34.0** | 34.5** | |
| (SE) |  |  | (3.54) | (3.55) | |
| **Volume of contract**  (per 100,000 doses) |  |  |  | -11.0** | |
| (SE) |  |  |  | (3.0) | |
| **Number of offers**  (both manufacturers) |  |  |  | -4.59* | |
| (SE) |  |  |  | (2.0) | |
| **Level of procurement** |  |  |  | 9.0** | |
| (SE) |  |  |  | (2.28) | |
| **GDP** |  |  |  | 0.000648** | |
| (SE) |  |  |  | (0.00027) | |
| **Contract Duration** |  |  |  | -2.07* | |
| (SE) |  |  |  | (0.85) | |
| **Award Criteria** |  |  |  | -2.25* | |
| (SE) |  |  |  | (1.91) | |
| **Random Effects and Model fit statistics** |  |  |  |  | |
| Variance Intercept | 121.2 | 108.7 | 117.6 | 62.06 | |
| (SD) | (11.01) | (10.43) | (10.8) | (7.88) | |
| Variance residual | 444.3 | 110.0 | 63.7 | 55.15 | |
| (SD) | (21.08) | (10.49) | (7.98) | (7.43) | |
| −2 Log Likelihood | 1622 | 1412 | 1334 | 1294 | |
| **p<0.01; *p<0.05, SE=Standard Error, SD= Standard Deviation, Number of obs: 178, groups: 50 | | | | |  |

1. Unit prices in Austria were calculated based on Equation 2. Number of births in Austria was from Statistics Austria available at [www.statistik.at](http://www.statistik.at). Vaccine coverage in Austria was 60% and was extracted from Borena W et al., Papillomavirus Research (2016). [↑](#footnote-ref-1)
2. For Iceland the tender for the HPV vaccine is a framework agreement. Framework agreements are awarded for certain goods and for a certain amount of time, without the exact quantities being known (volume/doses). Individual purchases are made after awarding of the framework agreement. Unit prices of the tender offers were identified on the website of Ríkiskaup (Central Public Procurement operating under the auspices of the Ministry of Finance of Iceland [**www.rikiskaup.is**](http://www.rikiskaup.is)**)**, using the identification code and name of the contract awards according to the TED documents. Winner of the tender and other parameters were collected from the Ted documents. Number of doses in Iceland was calculated based on Equation 1. Number of births was extracted from statistics Iceland, available at [www.px.hagstofa.is](http://www.px.hagstofa.is), vaccine coverage in Iceland is 90% according to Bruni et al., The Lancet Global Health (2016), and the dosing schedule for the first contract was set at 3 and set at 2 for the second contract dosing schedule, according to national recommendations. Contract duration was derived via personal communication. [↑](#footnote-ref-2)
3. The first tender was awarded for the vaccination of boys and the second tender for vaccination of girls. [↑](#footnote-ref-3)
4. The second tender award was “HPV vaccine for boys”. [↑](#footnote-ref-4)
5. The contract was for the 2013/2014 vaccination programme and the option of 1-year extension was used for the year 2014/2015 [↑](#footnote-ref-5)
6. In the tender of 2015 there was a separate tender for boys (No 37) that was awarded in October 2016. [↑](#footnote-ref-6)
7. Number of doses for this contract was estimated based on the previous contracts and the fact that this contract was for vaccination of preadolescent boys and girls. [↑](#footnote-ref-7)
8. The contract notice for 2012 found on the Ted website, but the end-value and number of doses was not reported. Unit prices were extracted from the list of the vaccine tariffs as were published on the website of the Dutch Healthcare Authority ([**www.nza.nl**](http://www.nza.nl)**).** Number of doses was calculated based on Equation 1. Vaccine coverage in the Netherlands was 60% and number of births obtained from Statistics Netherlands ([www.cbs.nl](http://www.cbs.nl)). Number of doses for 2012 and 2013 was set at 3 and after 2014 was set at 2, according to the national vaccination programme. Dose estimation was also compared to the number of doses reported in agreement of 2016 (TED document 2016/S 119-212098). This tender aimed for 120,000 doses per year. [↑](#footnote-ref-8)
9. Number of girls to be vaccinated for this contract was estimated based on previous and next contracts. [↑](#footnote-ref-9)
10. Although procurement procedure in Portugal is organised at national level, original contracts purchasing vaccines at national level were not located (attempts to get information via personal communication were not successful). However, using the identification code we located contracts/order forms from regional health authorities of Portugal, which contained unit prices and contract volume at regional level. These documents were collected from Base, the Portal of Public Contracts in Portugal ([**www.base.gov.pt**](http://www.base.gov.pt)**)**. Unit prices for the period 2008 until 2017 were extracted from the available contracts or purchase orders in Base platform, whereas the number of doses was estimated based on Equation 1. Number of births in Portugal was extracted from Statistics Portugal available at [www.ine.pt](http://www.ine.pt). Vaccination schedule was set at 3 doses and vaccine coverage was set at 90%.For contracts after 2014 vaccination schedule was set at 2 doses. [↑](#footnote-ref-10)
11. The HPV vaccine was introduced into the National Vaccination Programme Portugal in October 2008 with vaccination of 13 years old girls (cohort 1995) and a 3 dose schedule (0,2,6 months) (Source: Vacinação contra infecções por Vírus do Papiloma Humano (HPV), published on 27.05.2008 in Direção-Geral da Saúde (Directorate-General of Health), Lisbon, Portugal available at [www.dgs.pt](http://www.dgs.pt). Total end value of the contract and contract duration were extracted from the TED documents. Number of births in Portugal was extracted from Statistics Portugal available at [www.ine.pt](http://www.ine.pt). Vaccination schedule was set at 3 doses and vaccine coverage was set at 90%. From 2009 to 2011 the national vaccination campaign included a cohort of 17 year-old girls (born between 1992 and 1994). [↑](#footnote-ref-11)
12. Number of doses per year was estimated based on previous and subsequent contracts. [↑](#footnote-ref-12)
13. The end-value contract was retrieved from the Ted documents. Unit price was identified on the website of the [www.juntadeandalucia.es](http://www.juntadeandalucia.es) and number of doses for this contract was reported in “The social value of vaccines Elements of reflection to facilitate access, April 2015 (El valor social de las vacunas Elementos de reflexión para facilitar el acceso), a report about vaccine pricing in Spain prepared by Deloitte (located via the search process using the identification code of the agreement). [↑](#footnote-ref-13)
14. For the tender 123/2012 in Spain-Catalonia not all award documents were found. Hence, for number of offers received and the award criteria used, we assumed it to be equal to that of previous and next tenders. [↑](#footnote-ref-14)
15. Number of doses for this contract was estimated based on average number of doses from next contracts and the previous notice MS-CON1-10-008, which required 38,000 doses. [↑](#footnote-ref-15)
